# Supplementary material for: Seedless: on-the-fly pulse calculation for NMR experiments
Source: Nat Commun. 2025 Aug 7;16:7276. doi: 10.1038/s41467-025-61663-8 (PMC12332029; doi:10.1038/s41467-025-61663-8)
Supplement: Supplementary file 1 — Supplementary Information [file 41467_2025_61663_MOESM1_ESM.pdf]

Supplementary Information for

Seedless: On-the-fly pulse calculation for NMR experiments

Charles Buchanan[1,2], Gaurav Bhole [3,4], Gogulan Karunanithy[2,5], Virginia Casablancas-Antràs[1,2], Adeline Poh[6], Ben G. Davis[6], Jonathan A. Jones[3*], Andrew J. Baldwin[1,2,6*]

Contents:

Supplementary Note 1. Theoretical description of imaging experiments
Supplementary Note 2. Formal derivation of the Seedless formalism
Supplementary Note Table S2.1 A summary of the formulations used by Seedless
S2.1 Introduction
S2.2 Unitary propagators and the state-to-state fidelity
S2.3 State-to-state fidelity using product operators
S2.4 Propagator fidelities (universal rotations)
S2.5 Calculating fidelities and gradients
S2.6 XYcite pulses
S2.7 Suppression pulses
S2.8 Recipe of a Seedless calculation
S2.9 Exploitation of symmetry and implementation
S2.10 Appendix A: The average state of a single spin
S2.11 Appendix B: Explicit forms of X and G
S2.12 Appendix C: Products of 2×2 scaled-unitary matrices
Supplementary Note 3. Specific Pulse sequence design using Seedless
S.3.1 ^{13}C HSQC imaging
S.3.2 ^{19}F broadband 1D
S.3.3 ^{1}H perfect echo 1D
S.3.4 ^{15}N sensitivity enhanced HSQC
S.3.5 Sensitivity enhanced HNCO
S.3.6 Sensitivity enhanced HNCA
S.3.7 Sensitivity enhanced HNCACO
S.3.8 Sensitivity enhanced HNCOCA

Supplementary Note 4. Seedless operation instructions
Supplementary Note 5. Pulse performance comparisons
Table S5: Comparison of Seedless to pre-existing pulses.
S5.1 Genetics AI and Seedless (with Supplementary Note Figure 5.1)
S5.2 SURBOP and Seedless (with Supplementary Note Figure 5.2)
S5.3 GOODCOP and Seedless (with Supplementary Note Figure 5.3)
S5.4 OC1.2 pulses and Seedless (with Supplementary Note Figure 5.4)
S.5.5 Streamlined OC1.2 pulses generated by seedless (Supplementary Note Figure 5.5)

Supplementary References

Supplementary Fig. 1: Graphical overview of seedless pulses
Supplementary Fig. 2: B_1 inhomogeneity compensation revealed using imaging.
Supplementary Fig. 3: Detailed comparison of Seedless ^{19}F ultrabroadband experiment
Supplementary Fig. 4: Monitoring progress of a seedless optimisation.
Supplementary Fig. 5: Comparison of 600 MHz and 950 MHz ^{15}N HSQC data Seedless/rectangular
Supplementary Fig. 6: Cα-Cβ decoupling
Supplementary Fig. 7: Practical comparison on OC1.2 and seedless pulses.

Supplementary Note 1

Theoretical description of imaging experiments

To accompany **Main Text Fig. 1**, a brief description of the imaging experiment is presented. The imaging pulse sequence will lead to the following signal for a spin of gyromagnetic ratio γ, where the total acquisition time is a_t, the strength of the applied gradients is *G* and the position of the spin in the relevant axis is z

$$s(z,t) = e^{i\gamma Gz\, a_t/2} e^{i\gamma Gz\, t}.$$

We need to integrate this over the NMR tube, following weighing each slice by the projected spin density on the axis being integrated, $\rho(z)$,

$$S(t) = \int_0^\infty \rho(z) s(z,t) dz,$$

which we can then Fourier transform and take the absolute value to get an image:

$$I(\omega) = Abs\left[\int_{-\infty}^{\infty} S(t) e^{i\omega t} dt\right].$$

In the case where the distribution of spins ($\rho(z)$) is 1 between 0 and Z, and 0 elsewhere, then the image will be

$$I(\omega) = Abs\left[\int_{-\infty}^{\infty}\int_0^Z e^{i\gamma Gz\frac{a_t}{2}} e^{i\gamma Gz\, t} e^{i\omega t} dz dt\right] = Abs\left[\int_{-\infty}^{\infty} \frac{-2i\left(1 - e^{i\gamma G(a_t - 2t)Z}\right)}{\gamma G(a_t - 2t)} dt\right]$$

$$= \frac{\sqrt{\pi}}{2\gamma G} H(\omega, 0, \gamma GZ)$$

Where H is a rectangular function of ω, equal to 1 between 0 and γGZ, and 0 otherwise. In an experiment we will acquire *n* time points. The total length spanned by the points in the image will be

$$sw(cm) = \frac{2\pi n}{\gamma G a_t},$$

where the units of G are in G cm^{-1}, and the units of γ are rad G^{-1}s^{-1}. The spatial resolution, the distance spanned between two points in the image, will be sw/n. In **Main Text Fig 1B**, G was 12 G cm^{-1}, a_t was 2.56 ms for 493 points, and so the image spans 3.78 cm, with a spatial resolution of 0.077 mm.

Supplementary Note 2

Formal derivation of the Seedless formalism

Type	W	A	B	I	$\frac{dI}{dp_j}$
1. Universal/identity (S2.4 eq (20))	$\frac{1}{2}U^\dagger$	WV	$-A$	$1 - \mathrm{Tr}\,(A)$	$\mathrm{Re}\left[\mathrm{Tr}\left(BC_j\right)\right]$
2. state-to-state (ρ) (S2.2 eq (13))	$\rho_s V^\dagger \rho_f$	WV	$-2A$	$1 - \mathrm{Tr}\,(A)$	$\mathrm{Re}\left[\mathrm{Tr}\left(BC_j\right)\right]$
2. state-to-state (I) (S2.3 eq (16))	$I_s V^\dagger I_f$	WV	$-2A$	$\frac{1}{2} - \mathrm{Tr}\,(A)$	$\mathrm{Re}\left[\mathrm{Tr}\left(BC_j\right)\right]$
3. XYcite (I) (S2.6 eq (26))	$I_z V^\dagger I_z$	WV	$4\mathrm{Tr}\,(A)A$	$[\mathrm{Tr}\,(A)]^2$	$\mathrm{Re}\left[\mathrm{Tr}\left(BC_j\right)\right]$
4. suppression (I) (S2.7 eq (27))	$I_z X_k^\dagger I_z$	WX_k	$-2A_k$	$\frac{n}{2} - \sum_{k=1}^{n} \mathrm{Tr}\,(A_k)$	$\sum_{k=j}^{n} \mathrm{Re}\left[\mathrm{Tr}\left(B_k C_j\right)\right]$

Supplementary Note Table 2.1: A summary of the four restraints that can be imposed by Seedless on spins. For universal restraints, a target matrix U must be supplied or for state-to-state, starting (s) and finishing (*f*) states are required. A user supplies identifiers such as 90x or Iz -Iy, as described in the user instructions (**Supplementary Note 4**). In a Seedless calculation, for all offset frequencies and B_1 values the partial products X_j and C_j are calculated for each element of the pulse j. The target matrix *W* is then used to compute *A*, from which the infidelity value *I* and B are calculated. Finally, the derivatives are obtained for each element *j* from *B* and C_j. Here ρ indicates the use of density matrices and I indicates use of the traceless product operator matrices when formulating the restraint. While either could be used in principle for state-to-state calculations, we have implemented product operator matrices. The specific equations and sections that derive them are noted for ease of reference.

S2.1. Introduction

Seedless allows a user to specify a series of chemical shift 'bands', specified in PPM, together with other key properties such as spectrometer frequency, carrier position in PPM, peak B_1 field and pulse duration. The individual elements within the pulse are then optimized to produce a pulse that can perform these operations. Amplitudes and phases of elements can be optimized with Seedless, but as described in the text, outstanding pulses are produced keeping the amplitude constant and only varying the phases. The specific restraints for the four different types of transformation, universal, state-to-state, XYcite and suppression are

summarised (**Table 1**, **Supplementary Table 2.1)**, and then formally derived in the following sections. We conclude by identifying a number of critical symmetries that can be exploited in numerical calculations to provide the efficiencies we achieve with Seedless calculations [1,2].

The methods here are adopted from the GRadient Ascent Pulse Engineering (GRAPE) family of techniques in optimal control[3], and specialising the results to the case of single isolated spin $\frac{1}{2}$ nuclei. This allows analytic forms of the key expressions to be combined with low-level programming techniques to greatly speed up the conventional algorithm. Much of the material in this SI is entirely standard but is presented for the lay audience and to make our notation completely clear. We will in part follow the notation in the original GRAPE paper with slight modifications to give a more uniform presentation across the different cases we consider.

As described in the text, an imperfect pulse will take the starting state s to some state f', instead of the desired finishing state f. To assess the performance of the pulse, we need to evaluate the similarity of f and f' using a fidelity function. The fidelity is real-valued (so that it is possible to define an ordering), and ideally normalised to vary between 1 (when two states are identical) and 0 (when two states are as different as possible). In the most general case, when dealing with mixed states described by density matrices ρ, the correct expression is the Uhlmann-Jozsa fidelity[4] defined as

$$F = Tr\left(\sqrt{\sqrt{\rho'_f}\rho_f\sqrt{\rho'_f}}\right)^2 = Tr\left(\sqrt{\rho'_f\rho_f}\right)^2. \qquad (1)$$

The equivalence of the left and right hand sides of the equation was recently established[5]. In this work we will concentrate only on single isolated spins which can be described entirely in terms of pure states, allowing us to use either ket $|\psi\rangle$ or pure state density matrix $\rho = |\psi\rangle\langle\psi|$ descriptions. All density matrices ρ from here will be assumed to be proper normalised density matrices which correspond to pure states, and so are Hermitian $(\rho^\dagger = \rho)$ with unit

trace ($\mathrm{Tr}\,(\rho) = 1$) and idempotent ($\rho^2 = \rho$). For pure states, a simpler approach is possible. Defining the overlap integral

$$G = \langle \psi_f \mid \psi_f' \rangle \tag{2}$$

exactly reduces the Uhlmann fidelity to the pure state-to-state fidelity,

$$F = GG^* = \langle \psi_f \mid \psi_f' \rangle \langle \psi_f' \mid \psi_f \rangle = \mathrm{Tr}\left(|\psi_f'\rangle \langle \psi_f' \mid \psi_f \rangle \langle \psi_f| \right) = \mathrm{Tr}\left(\rho_f' \rho_f \right), \tag{3}$$

where $\rho = |\psi\rangle\langle\psi|$. The inter-conversion between the ket and density matrix formulations is permitted because of the invariance of the trace to cyclic permutation of a product of matrices. Note that the product of a row vector (a bra) by a column vector (a ket) gives a 1-by-1 matrix, rather than a scalar number, and it is necessary to take the trace of the final result to extract the desired result as a simple number, so there is an implicit trace operation in the ket based definition.

In practice it remains possible to use the pure state fidelity definition in some restricted but experimentally important special cases involving mixed states, most notably when comparing the effects of two different unitary transformations on the same state[6], but care should be taken to ensure that the simplified fidelity (**equation 3**) can be used instead of the most general form (**equation 1**).

In general, G will be complex, necessitating the use of the square modulus to give a real fidelity. We will show that in some special cases G will always be real, and so for 'propagator fidelities' or 'universal rotations' we can simplify further to $F = G$, where F is now strictly a 'square root' fidelity but remains a real number that cannot exceed 1 (**Supplementary Note 2.4**).

In all cases considered here, we will show that our fidelity or general cost function F can be rearranged into the form $F = \mathrm{Tr}\,(VW)$, where V is the unitary transformation described by the

pulse, and W is a target matrix, whose specific form varies depending on the type of restraint (**Table 1, Supplementary Table S2.1**).

In practical calculations, rather than maximising the fidelity, we will minimise the 'infidelity' I, a case specific cost function where our desired pulse is located at $I = 0$. If the fidelity function is correctly normalised, as it is for the state-to-state case constructed using 'proper' density matrices considered above, and for unitary propagators discussed below, the infidelity is simply $I = 1 - F$ (Supplementary Note **2.2**). If we instead use the traceless 'product operator' matrices (I_x,I_y,I_z) commonly used for NMR, this needs slight adaption to achieve an equivalent result (**Supplementary Table S.2.1**, **Supplementary Note 2.3**). We can integrate over all starting states to achieve a propagator fidelity, or universal rotation (**Supplementary Note 2.4**). As both cases conform to a general pattern, the analytic expressions for the infidelity and derivatives necessary for a GRAPE calculation elegantly fit within the same overall framework (**Supplementary Note 2.5**).

We will then treat two additional cases, 'XYcite' pulses (**Supplementary Note 2.6**) and 'water suppression' pulses (**Supplementary Note 2.7**), where we will construct alternative definitions using the state-to-state formalism (**Supplementary Notes 2.2/3)**. Finally, we identify unusual symmetries that arise in 2×2 matrices describing both the spin states and propagators for spin $\frac{1}{2}$ that allow us to implement a highly efficient computation method for both infidelities and gradients. These are essential for an effective search algorithm and together are responsible for the efficiency of the Seedless calculation (**Supplementary Notes 2.8/9**). Thus, while we are using a formalism based on density matrices implying matrix/matrix multiplication, the manipulations of all objects are reduced to 2×1 vector/vector multiplications, which is even more efficient than the matrix/vector multiplications expected for a purely ket-based calculation. Input files and pulses for all experiments discussed in this manuscript are provided with an associated download, and a manual describing how to setup an input file is available (**Supplementary Note 5**).

S2.2. Unitary propagators and the state-to-state fidelity

The Hamiltonian for an isolated spin $\frac{1}{2}$ nucleus in the presence of an RF field close to resonance can be written in the rotating frame as

$$\mathcal{H} = \omega_0 I_z + \alpha\big(\cos\,\phi I_x + \sin\,\phi I_y\big), \tag{4}$$

where ω_0 (the offset frequency) and α (the RF nutation rate, or B_1 amplitude) are both written in angular frequency units, so we are dropping a factor of $\hbar$ throughout and we are using the standard angular momentum matrices

$$I_x = \frac{1}{2}\begin{pmatrix} 0 & 1 \\ 1 & 0 \end{pmatrix},\ I_y = \frac{1}{2}\begin{pmatrix} 0 & -\mathrm{i} \\ \mathrm{i} & 0 \end{pmatrix},\ I_z = \frac{1}{2}\begin{pmatrix} 1 & 0 \\ 0 & -1 \end{pmatrix} \tag{5}$$

normalized so as to ensure that the familiar commutator relation $\left[I_{i,}I_j\right] = \mathrm{i}\epsilon_{ijk}I_k$where ijk are xyz and ϵ_{ijk} is the Levi-Civita symbol. We are simply dropping the counter rotating component of the RF field; a more careful analysis shows that the main effect of this is to produce a very small Bloch-Siegert frequency shift[7], subtly changing the offset frequency while the RF is applied. Note that it is only the true Bloch-Siegert shift, arising from the counter rotating component, which is neglected: the closely related phase shift arising from excitation far off-resonance, described by Ramsey, is automatically included[8]. This Hamiltonian is constant during a simple pulse of length t, and so the solution to the time-dependent Schrödinger equation for a pure state is given by

$$|\psi(\tau)\rangle = V|\psi(0)\rangle, \tag{6}$$

where because $\mathcal{H}$ is Hermitian, the propagator

$$V = \exp\left(-\mathrm{i}\mathcal{H}t\right) \tag{7}$$

is unitary, so that $VV^\dagger = V^\dagger V = E$, where

$$E = \begin{pmatrix} 1 & 0 \\ 0 & 1 \end{pmatrix} \tag{8}$$

is the identity operator. The evolution of the corresponding bra is given by

$$\langle\psi(\tau)| = \langle\psi(0)|V^{\dagger} \tag{9}$$

as the bra is the adjoint of the ket and the adjoint of a product is the product of the corresponding adjoints in reverse order, $(AB)^{\dagger} = B^{\dagger}A^{\dagger}$. A shaped pulse can be divided up into a number of segments, each of length t, with a fixed offset but with the amplitude (α) and phase (ϕ) different in each segment. We will later specialise to phase-only control, where the amplitude is the same in each pulse, but we begin with the more general case. For a pulse with n segments the combined propagator is given by the time-ordered product

$$V = V_n V_{n-1} \dots V_2 V_1, \tag{10}$$

of the sub-propagators for the individual segments

$$V_j = \exp\left(-\mathrm{i}\mathcal{H}_j t\right), \tag{11}$$

where

$$\mathcal{H}_j = \omega_0 I_z + \alpha_j\left(\cos\ \phi_j I_x + \sin\ \phi_j I_y\right) \tag{12}$$

as before. The effect of the pulse on some initial state is $|\psi_f'\rangle = V|\psi_s\rangle$, while the desired final state can be written as $|\psi_f\rangle = U|\psi_s\rangle$, where U is some unitary which exactly performs the desired operation for that starting state $|\psi_s\rangle$. Thus, the state-to-state fidelity can be rewritten as

$$F_s = \langle\psi_s|U^{\dagger}V|\psi_s\rangle\langle\psi_s|V^{\dagger}U|\psi_s\rangle = \mathrm{Tr}\left(U\rho_s U^{\dagger}V\rho_s V^{\dagger}\right) = \mathrm{Tr}\left(\rho_f V\rho_s V^{\dagger}\right) = \mathrm{Tr}(VW_s) \tag{13}$$

where $W_s = \rho_s V^{\dagger}\rho_f$. Note that although this expression is written in terms of two unitary operations, U and V, it is still a state-to-state fidelity because their effect is evaluated for the starting state $|\psi_s\rangle$.

S2.3. State-to-state fidelity using product operators

For isolated spin $\frac{1}{2}$ there is a direct mapping between pure states written in terms of the two kets that form the eigenbasis of the Hamiltonian describing the static field, $|\uparrow\rangle$ and $|\downarrow\rangle$, and the angular momentum operators, sometimes called one-spin 'product operators', that are typically used in NMR calculations,

$$I_z = \frac{1}{2}\begin{pmatrix} 1 & 0 \\ 0 & -1 \end{pmatrix} = \frac{1}{2}(|\uparrow\rangle\langle\uparrow| - |\downarrow\rangle\langle\downarrow|),\ \frac{1}{2}E = \frac{1}{2}\begin{pmatrix} 1 & 0 \\ 0 & 1 \end{pmatrix} = \frac{1}{2}(|\uparrow\rangle\langle\uparrow| + |\downarrow\rangle\langle\downarrow|), \quad (14)$$

and sums and differences produce

$$|\uparrow\rangle\langle\uparrow| = \frac{1}{2}E + I_z, \qquad |\downarrow\rangle\langle\downarrow| = \frac{1}{2}E - I_z, \quad (15)$$

The traceless Cartesian angular momentum operators when used to describe a density matrix such as I_z, are sometimes called 'deviation density matrices'[9] in the terminology of quantum information processing. Unlike pure state density matrices, they are not idempotent, and unlike proper mixed state density matrices they do not have trace one, but they are Hermitian and can with care be used to replace pure state density matrices.

The state-to-state fidelity in terms of density matrices (**equation 13**) for a single spin can then be written in terms of product operator matrices

$$\begin{aligned} F_s = \mathrm{Tr}\left(\rho_f \rho_f'\right) &= \mathrm{Tr}\left(U|\uparrow\rangle\langle\uparrow|U^\dagger V|\uparrow\rangle\langle\uparrow|V^\dagger\right) \\ &= \mathrm{Tr}\left(U\left[\frac{1}{2}E + I_z\right]U^\dagger V\left[\frac{1}{2}E + I_z\right]V^\dagger\right) \\ &= \mathrm{Tr}\left(U\left[\frac{1}{2}E\right]U^\dagger V\left[\frac{1}{2}E\right]V^\dagger + U\left[\frac{1}{2}E\right]U^\dagger V[I_z]V^\dagger + U[I_z]U^\dagger V\left[\frac{1}{2}E\right]V^\dagger + U[I_z]U^\dagger V[I_z]V^\dagger\right) \\ &= \frac{1}{4}\mathrm{Tr}\left(UU^\dagger VV^\dagger\right) + \frac{1}{2}\mathrm{Tr}\left(UU^\dagger VI_zV^\dagger\right) + \frac{1}{2}\mathrm{Tr}\left(UI_zU^\dagger VV^\dagger\right) + \mathrm{Tr}\left(UI_zU^\dagger VI_zV^\dagger\right) \\ &= \frac{1}{2}\mathrm{Tr}\left(\frac{1}{2}E\right) + \frac{1}{2}\mathrm{Tr}\left(VI_zV^\dagger\right) + \frac{1}{2}\mathrm{Tr}\left(UI_zU^\dagger\right) + \mathrm{Tr}\left(UI_zU^\dagger VI_zV^\dagger\right) \\ &= \frac{1}{2} + \mathrm{Tr}\left(UI_zU^\dagger VI_zV^\dagger\right) = \frac{1}{2} + \mathrm{Tr}\left(I_f I_f'\right), \end{aligned} \quad (16)$$

where we have used the linearity of the trace operation and the fact that unitary operators do not change the trace of a matrix. We may assume without loss of generality that the initial ket

is $|\uparrow\rangle$ as any other initial state can be handled by adjusting U and V. From this it follows that expressions such as $\mathrm{Tr}\left(I_f I_{f'}\right)$ can be used as if they were state-to-state fidelities $\mathrm{Tr}\left(\rho_f \rho_f'\right)$, except that the values now range from $-\frac{1}{2}$ to $+\frac{1}{2}$ instead of 0 to 1 and so the infidelity becomes $I = \frac{1}{2} - \mathrm{Tr}\left(I_f I_{f'}\right)$. State-to-state calculations in Seedless use product operator matrices.

Strictly speaking, we have referred to isolated spins thus far. The same approach can be used for thermal states of ensembles of single spins, which take the form

$$\rho = \frac{1}{2}E + pI_z \quad , \tag{17}$$

where the polarisation $p \sim 10^{-4}$ is the fractional population difference between the two states. This simply carries through the calculations as a scaling term, but as the polarisation is unchanged by unitary operations it is permissible to effectively pretend that $p = 1$. In the language of quantum information processing this is the observation that pseudo-pure states[10] are indistinguishable from pure states except for a scaling term, and that for single spins the thermal state is a pseudo-pure state. Generalising this approach to multiple spins is more complicated because the product operators no longer correspond to pure states[2], but that does not concern us here.

S2.4. Propagator fidelities (Universal rotations)

All the fidelities considered so far have been state-to-state fidelities because an initial state has always been specified, with the desired final state specified either explicitly or implicitly via the action of U on the initial state. For a universal rotation, which seeks to act correctly on every initial state, it is necessary to define a propagator fidelity. This can be derived by several different routes, but here we begin from **equation 2**, which neglects global phases, and write

$$G = \langle\psi|U^\dagger V|\psi\rangle = \mathrm{Tr}\left(U^\dagger V|\psi\rangle\langle\psi|\right) \tag{18}$$

for some initial state $|\psi\rangle$, and the average this over all initial states

$$\bar{G} = \mathrm{Tr}\left(U^{\dagger}V\overline{|\psi\rangle\langle\psi|}\right) \qquad (19)$$

where we have used linearity to take the averaging inside the operations. But the average of $|\psi\rangle\langle\psi|$ over all initial states is just $\frac{1}{2}E$ (**Supplementary Note 2.10**), and so

$$F_u = \bar{G} = \frac{1}{2}\mathrm{Tr}\left(U^{\dagger}V\right) = \mathrm{Tr}(W_u V) = \mathrm{Tr}(VW_u), \qquad (20)$$

where $W_u = \frac{1}{2}U^{\dagger}$. This 'propagator fidelity' expression is applicable for general rotors if global phases can be ignored. For the special case of an isolated single spin $\frac{1}{2}$ this is safe as long as the unitaries U and V are expressed in 'NMR terms', that is as exponentials of traceless Hamiltonians, in which case the combination $U^{\dagger}V$ will also be a unitary corresponding to a traceless Hamiltonian. For unitaries of this kind the two diagonal elements are always complex conjugates of each other, and so the trace is automatically real. Thus, the only possible global phases are $+1$ and -1, and maximising this fidelity, which runs over the range ± 1, will find the $+1$ solution. Like the 'true' fidelity (**equation 1**), this 'square root' fidelity F_u hence also runs from 0 to 1.

This residual global phase can cause problems in the analytic design of composite pulses[11] but we have never found it to cause difficulties for the numeric design of shaped pulses. It is, of course, possible to square this expression, thus avoiding any difficulties with global phases, and this can once again be related to an averaged state-to-state fidelity[12]. However, we prefer to use the simpler expression above because of its similarity to the state-to-state fidelities seen previously, all of which can be expressed as the trace of the product of two matrices.

In practical universal pulse calculations, the target transformation matrix U will for example take the form of $\mathrm{e}^{-\mathrm{i}\pi I_{\mathrm{x}}}$ for a 180°x unitary rotation or the identity when no chemical shift evolution is required. More generally $\mathrm{U} = \mathrm{e}^{-\mathrm{i}\theta n\cdot I}$ where *n* is a unit vector representing the axis of rotation, θ is the angle of rotation and the vector $I = I_x, I_y, I_z$, allowing pulses to be designed that can perform any desired rotation of the Bloch sphere.

S2.5 Calculating fidelities and gradients

Any optimisation routine will require the ability to calculate the chosen fidelity for any set of parameters and can be made much more efficient if it is possible to analytically calculate the gradient of the fidelity with respect to the parameters. We use the BFGS optimisation algorithm[13], which assumes that gradients are available, which are used to estimate properties of the Hessian (the matrix of second derivatives). Note that in optimal control problems of the kind considered here, the presence of false minima is not a significant problem[14], and so using rapid gradient-based approaches is sensible. We emphasise that we have never observed a Seedless optimisation becoming stuck in a false minimum.

The key breakthrough in the GRAPE family of approaches is the realisation that storing partial results allows gradients to be calculated efficiently[3], greatly speeding up the search process. For the special case of the single isolated spin $\frac{1}{2}$ this process can be further sped up by using analytic formulae to calculate the underlying terms and by performing matrix operations efficiently. Restricting the problem to varying only the pulse phases gives particularly simple results.

Our optimisations seek to find the set of amplitudes and phases that minimise the infidelity I, which in all cases is expressed in terms of the fidelity cost function $F = \mathrm{Tr}\,(VW)$. The notation used here differs slightly from that in the original GRAPE paper[6], but the underlying concepts are identical. To obtain derivatives of the fidelity with respect to individual element phases and amplitudes (p_j can be either α_j or ϕ_j) requires use of the product rule

$$\frac{\partial F}{\partial p_j} = \mathrm{Tr}\left(\frac{\partial W}{\partial p_j} V\right) + \mathrm{Tr}\left(W \frac{\partial V}{\partial p_j}\right). \tag{21}$$

The next stage is to expand V as a time ordered product and use the product rule again but noting that $\partial V_k / \partial p_j = 0$ unless $k = j$. Thus

$$\frac{\partial V}{\partial p_j} = V_n V_{n-1} \dots \left[\frac{\partial V_j}{\partial p_j}\right] \dots V_2 V_1 = X_n X_j^{\dagger} G_j X_{j-1} = X_n C_j, \tag{22}$$

where we define the partial products $X_j = V_j \dots V_2 V_1$ such that $V = X_n, C_j = X_j^{\dagger} G_j X_{j-1}$ and the derivative of matrix element j is $G_j = \partial V_j / \partial p_j$.

These forms allow gradients to be rapidly calculated once the sub-propagator gradients G_j are known. Explicit forms are given (**Supplementary Note 2.11**), where it is shown[15] that when $p_j = \phi_j$ then $G_j = \mathrm{i}[V_j, I_z]$. For universal rotations fidelities, $W_u = \frac{1}{2} U^{\dagger}, F_u = \mathrm{Tr}\,(W_u X_n)$, and $\partial W_u / \partial p_j = 0$, and so

$$\frac{\partial F_u}{\partial p_j} = \mathrm{Tr}\,(B_u C_j), \tag{23}$$

where $B_u = W_u X_n$. For the state-to-state fidelity, $W_s = \rho_s V^{\dagger} \rho_f, F_s = \mathrm{Tr}\,(W_s X_n)$, and so

$$\frac{\partial F_s}{\partial p_j} = \mathrm{Tr}\left(\rho_s \frac{\partial V^{\dagger}}{\partial p_j} \rho_f V\right) + \mathrm{Tr}\left(\rho_s V^{\dagger} \rho_f \frac{\partial V}{\partial p_j}\right). \tag{24}$$

As ρ_s and ρ_f are Hermitian, the two terms are complex conjugates of each other and so

$$\frac{dF_s}{dp_j} = 2\mathrm{Re}\left[\mathrm{Tr}\left(W_s \frac{dV}{dp_j}\right)\right] = \mathrm{Re}\left[\mathrm{Tr}\,(B_s C_j)\right], \tag{25}$$

where $B_s = 2 W_s X_n$. The same approach can be used for state-to-state fidelities defined in terms of product operators (deviation density matrices, **Supplementary Note 2.3, Supplementary Table S2.1**).

This approach can be extended to infidelities, if these are carefully defined with the correct range of fidelity values; the main effect is simply that the gradient changes sign as we are now seeking a minimum infidelity rather than a maximum fidelity. The method can also be applied to excitation pulses, where it is more natural to define the infidelity directly. In each case the (in)fidelities depend on terms of the form $\mathrm{Tr}\,(A)$, where $A = \mathrm{V}W$, and the gradients on terms of the form $\mathrm{Re}\left[\mathrm{Tr}\,(BC_j)\right]$, where the matrices A and B depend on the precise problem being solved, but C_j always takes the same form (summarised in **Supplementary Table S2.1)**. We

will now deal with two special cases of state-to-state pulses that may provide useful transforms in NMR experiments, XYcite and suppression.

S2.6. XYcite pulses

XYcite pulses are a special case of state-to-state pulses, being designed to take a single spin initially along the z-axis into the xy-plane, but without caring where in the plane a spin ends up, allowing this to vary for spins with different offset frequencies. Equivalently they take the state I_z into some combination of I_x and I_y, but with no attempt to specify what that combination is. None of the fidelities above are suitable for this purpose, as they all require either a final state or an explicit transformation to be specified. Instead, the appropriate target is to have no component along the z-axis at the end of the process. This can be achieved by using the following infidelity

$$I_{\mathrm{ex}} = \left[\mathrm{Tr}\left(I_z V I_z V^{\dagger}\right)\right]^2 = \left[\mathrm{Tr}\left(W_{\mathrm{ex}} V\right)\right]^2 \ , \qquad (26)$$

where $W_{ex} = I_z V^{\dagger} I_z$, giving an infidelity which must be minimised. It is essential to use the squared form here, as simply minimising $\mathrm{Tr}\,(W_{\mathrm{ex}}\, V)$ will find an inversion pulse, which takes I_z to $-I_z$. The derivative follows from **equation 23** with $B = 4\mathrm{Tr}\,(W_{\mathrm{ex}} V) W_{\mathrm{ex}} V$.

Excitation pulses of this type could be used, for example, in a heteronuclear INEPT sequence where we bring magnetisation from Z to the XY plane, leave it for a period, then return it back to z. In this case, the pulses will be used with a pair that has been time reversed and phase inverted together with a central $180°$ element designed to refocus chemical shift. While each spin will accrue an effectively random phase following the first XYcite pulse, the action of XYcite,180,Time/PhaseReversedXYcite will perfectly refocus the initial random phase, and together restore the spin to the positive z axis[16].

S2.7. Suppression pulses

When performing $^{1}\mathrm{H}$ NMR experiments in $\mathrm{H_2O}$ it is almost always necessary to apply one or more forms of water suppression. The sample of interest might be at a concentration on the order of $\mu\mathrm{M}$, and water at a concentration of $55\ \mathrm{M}$ will give roughly 10^8 times more signal than the sample.

All the fidelity formulations considered thus-far consider only the overall action of a pulse or the final state of the spin. It is easy to set constraints on the water band, either performing no overall rotation, or more simply taking I_z back to I_z at the end of the pulse. However, this does not give particularly effective water suppression, both because of the huge size of the water signal and also because of effects such as radiation damping which mean that the behaviour of the state is not well described by the assumed unitary evolution. The efficiency of our formulation of the optimisation allows us to consider a new form of suppression pulse, one that aims not just to 'not excite' water by the end of the pulse, but also aims as far as possible to avoid exciting water at each individual step of the pulse. The additional restraint can easily be implemented using the framework we have developed.

For the band where we seek to suppress water, we add to the fidelity a term which simply monitors the state of the water after each of the n steps of the pulse

$$F = \sum_{k=1}^{n} \mathrm{Tr}\left(A_k\right), \tag{27}$$

where $A_k = W_{miss,k} X_k$ and $W_{miss,k} = I_z X_k^{\dagger} I_z$. This contributes to the infidelity derivatives as

$$\frac{\partial I}{\partial \phi_j} = \sum_{k=j}^{n} \mathrm{Re}\left[\mathrm{Tr}\left(B_k C_j\right)\right], \tag{28}$$

which reflects the fact that a change in the j th sub-propagator will affect the state of the water at every point later in time. Here $B_k = 2W_{miss,k} X_k$.

When expressed in this form, the elements to incorporate the new fidelity adds significantly to the cost of the calculation. All the elements for A_k are calculated as part of the conventional end of pulse restraints, and so the extra cost comes from the additional loop required for the gradients in **equation 28**. As the individual elements C_j were also already calculated for the standard calculation, the cost comes from the fact that we need $n^2/2$ additional matrix products, rather than the n required where we apply the restraint only at the end of the calculation, leading to an additional multiplicative cost of $n/2$ in the number of matrix products when we use this restraint. While this calculation is expensive, the efficiency gains in our implementation are great enough that including this restraint remains practical.

S2.8 Recipe of a Seedless calculation

We have described schemes for imposing restraints of four types (**Table 1, Supplementary Table S2.1**): universal rotation, state-to-state, XYcite and suppression. In all cases, the infidelity for a single spin requires evaluation of $\mathrm{Tr}\,(A)$ where $A = \mathrm{V}W$ and the gradients for each pulse element j through $\mathrm{Re}\left[\mathrm{Tr}\,(BC_j)\right]$. The clear structural similarities between the restraint types allow extensive overlap in the underlying code.

In each case, a single function call to the fidelity/gradient calculation requires three parts. The first task is to loop over the n steps of the pulse to calculate and store X_j and C_j for each element. The second, the only case specific part, is to calculate the matrix A from which the cost function, the infidelity, I can be determined, and a second matrix for the derivatives, B. The final task is to loop over all elements of the pulse once more to calculate the gradients via the real part of $\mathrm{Tr}(BC_j)$. Only the first and third loops lead to computational expense as they require a number of matrix multiplications that scale linearly with the number of elements in the pulse, and these two are identical for all restraints. In the case of a 'suppression' pulse, the second and third tasks are repeated for 'subpulses' of length 1 to n leading to the third task scaling as $n^2/2$ rather than n. The schemes are summarised (**Table I** and **Supplementary Table S2.1)**.

To optimise a pulse, a series of 'bands' of chemical shift are specified together with a number of spins S whose resonance frequencies ω_s are linearly spaced within the limits of the band. For each band, each spin receives a restraint, Γ_s, one of the 4 types (**Supplementary Table 2.1**). In the case of a universal pulse, a value for U has to be specified for the band, which typically will be either $90^\circ_x = \mathrm{e}^{-\mathrm{i}\frac{\pi}{2}I_x}$, or $180^\circ_x = \mathrm{e}^{-\mathrm{i}\pi I_x}$, or the identity. For a state-to-state calculation, the initial and final states, I_s and I_f need to be specified, chosen from (I_x,I_y,I_z). For the excitation and suppression restraints, it is sufficient to indicate the presence of the restraint. The software will then determine what is required (**Table I**).

To render the pulses tolerant to B_1 inhomogeneity, we further define a set of factors b_r containing R elements, and weighting constants d_r, such that the field 'seen' by a spin during element j is $\alpha_j = f_j b_r \omega_R$ where f_j is a fractional amplitude for each pulse element (set to 1 in a 'phase only' calculation), ω_R is a constant peak field set for the calculation and b_r is the inhomogeneity factor where $b_r = 1$ corresponds to the action of the pulse exactly at the specified ω_R. We find that specifying three values, $b_r = 0.95, b_r = 1$, and $b_r = 1.03$, weighted $d_r = \frac{1}{4}, \frac{1}{2}, \frac{1}{4}$ respectively, will account for a typical variation in B_1 inhomogeneity found in an NMR spectrometer[17], but it is straightforward to include a larger number of values and/or to sample a wider range of inhomogeneities if desired. Noting explicitly that the infidelity of each spin in the calculation will be a function of the restraint type, Γ_s, the offset frequency of the spin, ω_s, and the field inhomogeneity factor, b_r, and further assuming $\sum d_r = 1$, then the overall global infidelity delivered to the optimiser is

$$\langle I \rangle = \frac{1}{S} \sum_s \sum_r d_r I(\Gamma_s, \omega_s, b_r), \tag{29}$$

where $\langle I \rangle = 0$ when all restraints are perfectly satisfied. In practice this is typically impossible for reasonable pulse lengths, so the goal of a calculation is a ‘tolerable’ value. Loosely, if the response function of the pulse, simulated at several B_1 values, ‘looks good’, then the performance at the spectrometer is likely to ‘be good’.

When incorporating the suppression constraint it is appropriate to scale its contribution by an additional factor γ to balance the fact that each missed restraint provides *n* contributions to a fidelity score per spin whereas all other restraints contribute just once. Intuitively increasing γ leads to better water suppression at the sacrifice of excitation, and a smaller γ does the reverse (Supplementary Note **.3.3**). Given that there are M spins of chemical shift ω_m in the 'suppression' band then the overall infidelity will receive an additional contribution of

$$\langle I_{\text{miss}} \rangle = \frac{1}{M} \sum_m \sum_r d_r I(\omega_m, \Gamma_s = \text{miss}, b_r). \quad (30)$$

And so overall

$$\langle I_{\text{total}} \rangle = \frac{S\langle I \rangle + \gamma M \langle I_{\text{miss}} \rangle}{S + \gamma M}.$$

As every individual sub-pulse will have some effect on the water spins, it is essentially impossible for $\langle I_{\text{miss}} \rangle$ to be reduced to zero, and it is particularly hard to minimise in the case of pulses where we desire two excitation bands that straddle a 'miss' region as described in the text (**Supplementary Note 3.3**). For this reason, $\langle I_{\text{miss}} \rangle$ is better thought of as a penalty function, which one seeks to reduce, rather than a ‘true’ infidelity as used in quantum information theory that needs to be reduced to zero for functional performance. Care is required with setting both γ and the RF field strength ω_R to obtain pulses with values of the combined infidelity that are 'low enough' to be useful.

S2.9 Exploitation of symmetry and implementation

Substantial reductions in calculation time are obtained from exploiting the underlying symmetry in the many 2×2 matrices that need to be generated and multiplied during a calculation. Every 2×2 unitary matrix corresponding to a traceless Hamiltonian has the general form

$$U = \begin{pmatrix} u_a & u_b \\ -u_b^* & u_a^* \end{pmatrix}, \quad (31)$$

and so is characterised by just two complex numbers, with four degrees of freedom. This observation is closely related to the use of quaternions to describe propagators in spin-$\frac{1}{2}$ systems[18]. If U actually is unitary then $U^{\dagger}U = UU^{\dagger} = E$, reducing the number of degrees of freedom to three, but the definition can be conveniently extended to 'scaled-unitaries' P, which have the general form of **equation 31** but with the weaker constraint that $P^{\dagger}P = PP^{\dagger} = c^2E$ for some real number c. Note that by this definition unitary matrices are a special case of scaled-unitary matrices with $c = 1$.

It can be shown by direct calculation (**Supplementary Note 2.12**) that the product of two scaled-unitary matrices is itself a scaled-unitary. Deviation density matrices such as I_z are not of scaled-unitary form but can be made so by multiplying them by $\pm$ i. True density matrices cannot be easily converted to this form, and so we choose to carry out state-to-state calculations using deviation density matrices, corresponding to conventional NMR notation to exploit this symmetry.

From this it follows that V_j, G_j, C_j and all other matrices obtained in our calculation are scaled-unitaries. We take advantage of two properties that arise from this. Firstly, the trace of a scaled-unitary matrix is always real, reducing the cost of a real product of two complex matrices (where we only concern ourselves with the diagonal elements in both cases) by a factor of 2. Furthermore, we need only the first row of the left-hand matrix (B), and the first column of the right-hand matrix (C_j), further reducing the memory requirements and matrix operations required to calculate the various quantities, making this product a vector/vector multiplication rather than matrix/matrix increasing the saving to a factor of 4. Secondly, all 2×2 matrix products can be replaced by matrix/vector products. If $W = XV$, then we can obtain the first column of W as a vector, W', by multiplying X by the first column of V, or $W' = XV'$. We can always reconstruct the form of W from its first column using **equation 31**. In this fashion, in part 1 of the calculation requires only the first column of X_j and C_j, part 2 requires only the first row of B, and the gradient calculations require only the real part of vector/vector

multiplication $\mathrm{Re}\left[\mathrm{Tr}\left(BC_j\right)\right]$. This implementation requires fewer operations than an equivalent formulation to obtain the infidelity explicitly using kets.

The code is written in $\mathrm{C}++$ and relies on pointers so that the machine code performing the matrix products contains the smallest number of CPU operations possible. The optimiser itself is a BFGS gradient descent algorithm[19-21] as previously established as a good method for this type of calculation[22].

S2.10 Appendix A: The average state of a single spin

In **Supplementary Note 2.4** we asserted that the effect of averaging the density matrix $|\psi\rangle\langle\psi|$ over all initial states ψ is simply $\frac{1}{2}E$. This result is obvious by symmetry, but a more explicit calculation is included below for completeness. An arbitrary pure state of a single isolated spin can be written in the standard basis as

$$|\psi\rangle = \alpha|\uparrow\rangle + \beta|\downarrow\rangle,$$

with the constraint that $|\alpha|^2 + |\beta|^2 = 1$. As quantum states are invariant under global phase changes, it is convenient to choose coefficients such that α is real and positive. These requirements are easily met by writing

$$|\psi\rangle = \cos(\theta/2)|\uparrow\rangle + \mathrm{e}^{\mathrm{i}\phi}\sin(\theta/2)|\downarrow\rangle,$$

with $0 \leq \theta \leq \pi$ and $0 \leq \phi < 2\pi$, corresponding to spherical polar coordinates. Thus, any quantum state can be mapped to a point on the surface of the unit sphere (the Bloch sphere). The average state is clearly a mixed state, and so must be described using a density matrix. This is obtained by averaging $|\psi\rangle\langle\psi|$ over the surface of the sphere

$$\bar{\rho} = \frac{1}{4\pi}\int_0^{\pi}\int_0^{2\pi}\begin{pmatrix}\cos^2[\theta/2] & \mathrm{e}^{-\mathrm{i}\phi}\cos[\theta/2]\sin[\theta/2] \\ \mathrm{e}^{\mathrm{i}\phi}\cos[\theta/2]\sin[\theta/2] & \sin^2[\theta/2]\end{pmatrix}\sin\theta\,\mathrm{d}\theta\mathrm{d}\phi = \begin{pmatrix}\frac{1}{2} & 0 \\ 0 & \frac{1}{2}\end{pmatrix},$$

giving $\frac{1}{2}E$ as required.

Another way to approach this begins from the observation that for a unitary transformation the pure state fidelity for any initial state $|\psi\rangle$ will always be the same as that for the state $|-\psi\rangle$ which lies directly oppose $|\psi\rangle$ on the Bloch sphere. In the language of quantum information processing this state is usually called $|\psi^{\perp}\rangle$ in the general case[23], although the sign notation is commonly used for states such as $|\pm z\rangle$ which lie along the cardinal axes of the Bloch sphere. The spherical average can be replaced by averaging over the six cardinal points[12], and as $\frac{1}{2}(|z\rangle\langle z| + |-z\rangle\langle -z|) = E$, and so on, this once again leads to the same result.

S2.11 Appendix B: Explicit forms of X and G

The Hamiltonian for a single spin-$\frac{1}{2}$ at offset ω_0 during an individual RF pulse of phase ϕ and amplitude α is

$$\mathcal{H} = \omega_0 I_z + \alpha\big(\cos\ \phi I_x + \sin\ \phi I_y\big),$$

with the associated propagator describing evolution for a time t given by $V = \exp\left(-\mathrm{i}\mathcal{H}t\right)$. In our calculations, a fixed field ω_R is specified, and an element specific scaling factor f is optimised. Moreover, when accounting for amplitude inhomogeneity, the calculation is repeated with different relative fields described by an overall scaling factor r, such that $\alpha = \omega_R f r$. The explicit form of V is easily evaluated. Defining $\Omega = \sqrt{\frac{1}{2}\mathrm{Tr}(\mathcal{H}^{\dagger}\mathcal{H}\,)} = \frac{1}{2}\sqrt{\omega_0^2 + \alpha^2}$:

$$\begin{aligned} V &= \cos\left(\Omega \mathrm{t}\right)E - \frac{\mathrm{i}}{\Omega}\sin\left(\Omega \mathrm{t}\right)\mathcal{H} \\ &= \begin{pmatrix} \cos\left(\Omega\mathrm{t}\right) - \mathrm{i}\dfrac{\omega_0}{2\Omega}\sin\left(\Omega\mathrm{t}\right) & -\mathrm{i}\dfrac{\alpha}{2\Omega}\cos\ \phi\sin\left(\Omega\mathrm{t}\right) - \dfrac{\alpha}{2\Omega}\sin\ \phi\sin\left(\Omega\mathrm{t}\right) \\ -\mathrm{i}\dfrac{\alpha}{2\Omega}\cos\ \phi\sin\left(\Omega\mathrm{t}\right) + \dfrac{\alpha}{2\Omega}\sin\ \phi\sin\left(\Omega\mathrm{t}\right) & \cos\left(\Omega\mathrm{t}\right) + \mathrm{i}\dfrac{\omega_0}{2\Omega}\sin\left(\Omega\mathrm{t}\right) \end{pmatrix} \end{aligned}$$

The derivative with respect to phase is

$$
\begin{aligned}
G^{\phi} = \frac{\partial V}{\partial \phi} &= \begin{pmatrix} 0 & -\frac{\alpha}{2\Omega}\cos\phi\sin(\Omega t) + \mathrm{i}\frac{\alpha}{2\Omega}\sin\phi\sin(\Omega t) \\ \frac{\alpha}{2\Omega}\cos\phi\sin(\Omega t) + \mathrm{i}\frac{\alpha}{2\Omega}\sin\phi\sin(\Omega t) & 0 \end{pmatrix}. \\
&= \mathrm{i}[V, I_z]
\end{aligned}
$$

Note $G(0,1) = -\mathrm{i}V(0,1)$ and $G(1,0) = \mathrm{i}V(1,0)$, allowing G^{ϕ} to be easily constructed from V. It is easily seen that G is a scaled-unitary, while V is obviously a unitary by construction.

The derivatives with respect to amplitude can also be evaluated analytically but are significantly more complicated and are not shown here. Once again, this derivative is a scaled-unitary. Seedless contains an option for amplitude modulation, but this is not used for any of the work shown here.

S2.12 Appendix C: Products of 2×2 scaled-unitary matrices

A pulse optimisation calculation treating the system as a single spin- $\frac{1}{2}$ requires many multiplications of scaled-unitary matrices. The time taken for the calculation can be significantly reduced by explicitly considering the structure of these matrices. The properties will be derived here. If $W = UX$, and both U and X are parameterised by two complex numbers then

$$
W = UX = \begin{pmatrix} u_a & u_b \\ -u_b^* & u_a^* \end{pmatrix} \begin{pmatrix} x_a & x_b \\ -x_b^* & x_a^* \end{pmatrix} = \begin{pmatrix} u_a x_a - u_b x_b^* & u_a x_b + u_b x_a^* \\ -u_b^* x_a - u_a^* x_b^* & -u_b^* x_b + u_a^* x_a^* \end{pmatrix}.
$$

It is clear that W is also a scaled-unitary matrix (**equation 31**). As the matrix contains only two independent complex numbers, we do not need to store the full 2×2 form. If we instead store only the first column (indicated by a prime ') then

$$
W' = UX' = \begin{pmatrix} u_a & u_b \\ -u_b^* & u_a^* \end{pmatrix} \begin{pmatrix} x_a \\ -x_b^* \end{pmatrix} = \begin{pmatrix} u_a x_a - u_b x_b^* \\ -u_b^* x_a - u_a^* x_b^* \end{pmatrix}.
$$

Thus, all matrix multiplications can be replaced by matrix/vector multiplications, halving both the number of required multiplications and the storage memory requirements. In cases where

the full matrix is required, the second column can be easily calculated from the first column. Moreover, in the case where the trace of a product is required,

$$\begin{aligned}\mathrm{Tr}\,(UX) &= u_a x_a - u_b x_b^* - u_b^* x_b + u_a^* x_a^* \\ &= (u_a x_a + u_a^* x_a^*) - (u_b x_b^* + u_b^* x_b) \\ &= 2\,\mathrm{Re}(u_a x_a) - 2\,\mathrm{Re}(u_b x_b^*)\,.\end{aligned}$$

Using a double prime to indicate the first row of a matrix, $U'' = (u_a u_b)$, gives

$$\mathrm{Tr}\,(UX) = 2\mathrm{Re}\,(U''X')$$

and we need only do a vector/vector multiplication, including an implicit trace. Finally, as the trace of the product of two scaled-unitary matrices will always be real, it is not necessary to explicitly take the real part. This result is used most notably when calculating the gradients where we only retain the top row of B_n, which we combine with the factor of 2, and the first column of C_j.

Supplementary Note 3

Specific Pulse sequence design using Seedless

The 8 sequences that were optimised for Seedless are individually discussed. Open and filled rectangles correspond to 90° and 180° degree pulses respectively. Blue filled rectangles correspond to zero-time 'identity' pulses, which ensure the final state is the same as the initial state, effectively preventing evolution due to J coupling and chemical shift. All triple resonance sequences were based on pulse sequences from the laboratory of Lewis Kay[24] (see **methods**). In the following figures, the performance of the pulse is shown together with the profile. For performance, magnetization is started on either the X, Y or Z positions, the pulse is applied, and the final locations (X,Y,Z) are indicated. This is repeated for three values of relative peak amplitudes of, 0.95, 1.00, 1.03. The traces for all three amplitudes are shown on each plot showing the performance of the pulse. Where individual traces for the amplitudes cannot be easily resolved visually, the performance of the pulse can be considered highly tolerant to amplitude fluctuation. The panels from the following figures were adapted from the reports automatically generated by Seedless upon each run (examples in **Supplementary Note 4**). ***Each of the following sections starts with a graphical pulse sequence with description, followed by detailed descriptions of each of the seedless pulses used to implement them.***

S.3.1 ^{13}C HSQC imaging

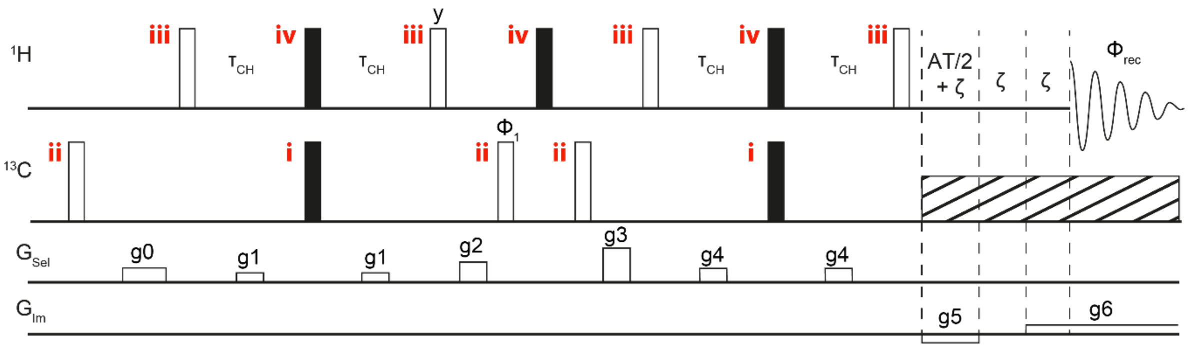

Shown in **Main Text Fig. 1**, applied to a sample of ^{13}C methanol where Φ_1 = (0,2) and Φ_{rec} = (0,2), gradient durations g0, g1, g2, g3 and g4 were 1 ms, 500 μs, 500 μs, 300 μs and 150 μs

with levels set to at 12.5%, 12.5%, 31.25%, 62.25% and 28.125% of the maximum value, respectively, to de-phase off-pathway magnetization. AT refers to acquisition time (set here to 2.56 ms), and ζ was set to 20 μs. For the imaging gradients, g5 and g6 were set at 6.25% for the durations specified. Two probes were used, a 5 mm 5 channel HCN probe where selection (G_{Sel}) and imaging (G_{Im}) gradients were both on the Z gradient channel (**Supplementary Fig. 2**). On the XYZ probe (**Main Text Fig. 1**) the two gradient channels could be separated. When G_{Sel}=Z , G_{Im} = X/Y and G_{Sel}=X, G_{Im}=Z (**Fig 1a**). The decoupler was applied throughout the imaging gradients to prevent unwanted evolution of ^{13}C scalar coupling, using a waltz16 pattern at a field of 5,882 Hz. The images are presented in 'absolute value' mode (theory in **Supplementary Note 1**).

Spectra were acquired on two different room temperature probes. **Main Text Fig. 1a:** a 5mm triple resonance probe equipped with xyz-gradients, **Supplementary Fig. S2:** a 5mm room temperature HCN probe with Z gradients, both at 600 MHz. Both were recorded with 492 complex points and a sweep width of 192,308 Hz. The interscan delay was 2s and 64 transients were recorded per FID for a total acquisition time of 2 minutes. The C-H inept was performed with τ_{CH}=0.00176 s, consistent with maximum coherence transfer J=142 Hz, the value for the molecule of interest and the recovery delay ζ was set to 200 μs. The spectra were manually processed using nmrPipe using an offset sine-bell apodisation function.

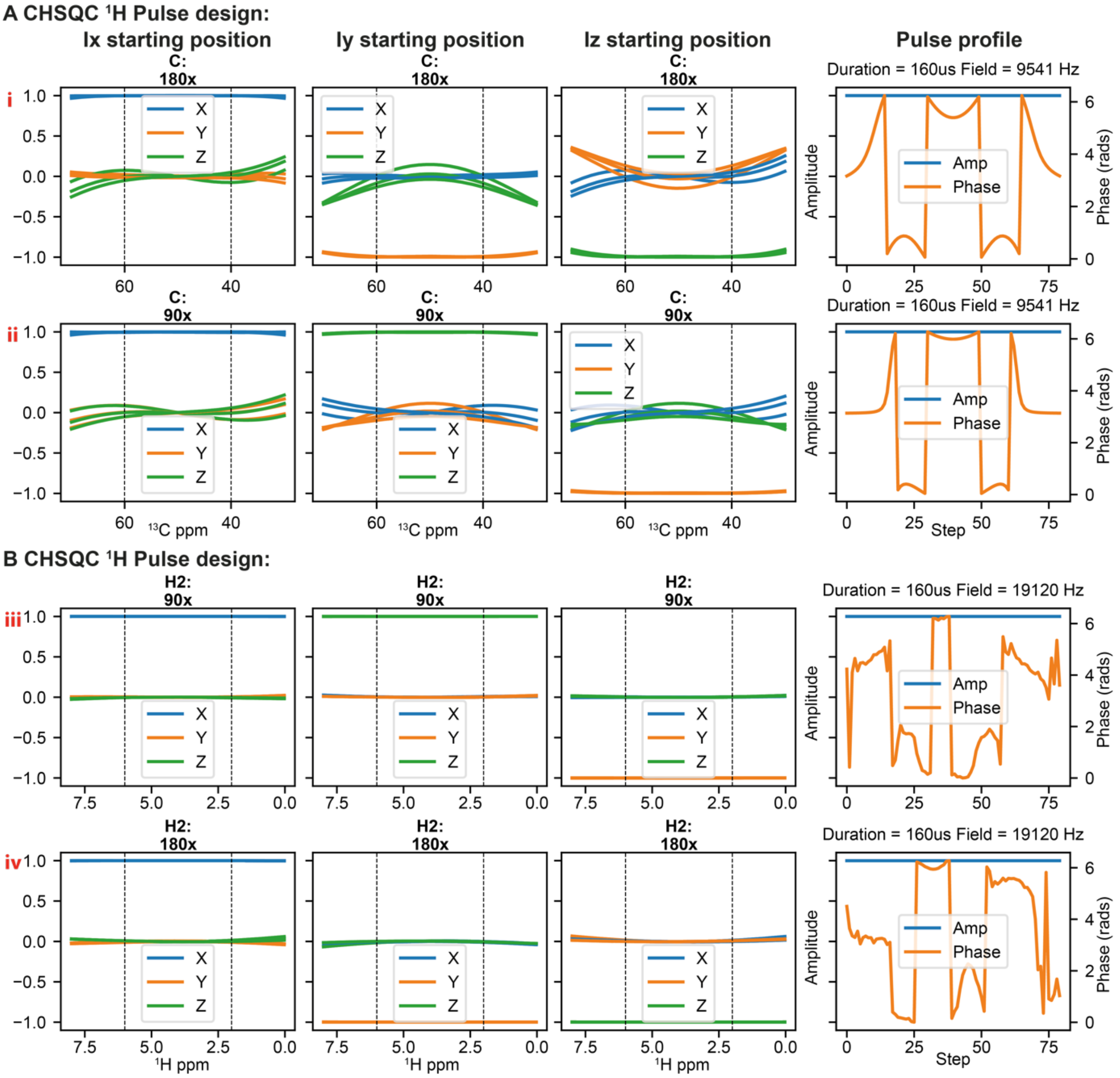

The ^{1}H and ^{13}C used were designed for imaging a solution of ^{13}C labelled methanol (**Main Text Fig. 1, Supplementary Fig. 1**), and the carrier frequencies were set to match the observed resonances (4.77 ppm (^{1}H) and 51.7 ppm (^{13}C)). The ^{13}C carbon pulses had a 20 ppm bandwidth that more than covered the 2.5 ppm range needed. The duration of all pulses was set to 160 μs, and all were of unitary type as stated, optimized in the specified regions shown. All pulses were applied at maximum B_1. Two room temperature probes were used. For a 5 mm room temperature three channel probe with XYZ gradients operating at 600 MHz: ^{1}H=19.1 kHz and ^{13}C=9.541 kHz (the pulses shown were optimized for this probe), and for a 5 mm room temperature three channel HCN probe operating at 600 MHz: ^{1}H=31.25 kHz and ^{13}C=17.361 kHz.

S.3.2 Broadband excitation ^{19}F 1D

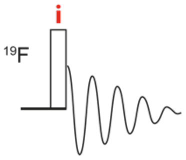

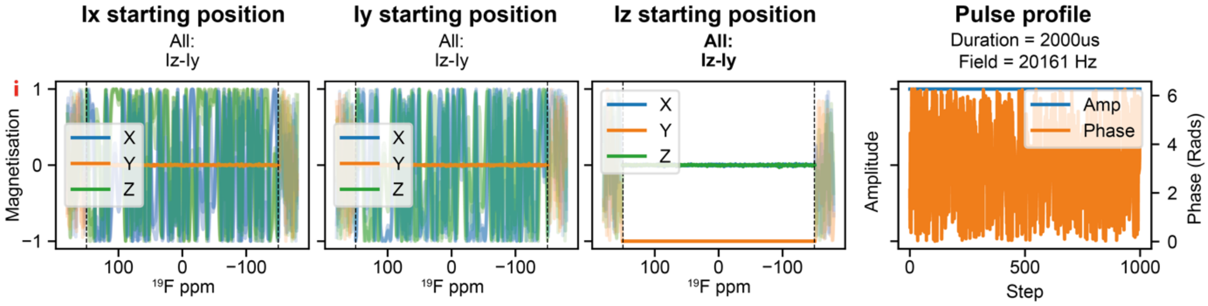

The simplest possible NMR pulse sequence. A Seedless $\mathrm{Z} \rightarrow -\mathrm{Y}$ excitation pulse was used with a B_1 field of 20.2 kHz and duration 2 ms ^{19}F spectra (**Main Text Fig. 1b**) in ca. 10 s (**Supplementary Fig. 4**) was recorded on a Bruker AVANCE NEO 600 MHz spectrometer with CPRHe-QR-1H/19F/13C/15N-5mm-Z helium-cooled cryoprobe. 681,818 complex points were recorded with a sweep width of 340,909Hz. The interscan delay was 10s and 4 transients per FID were recorded for a total acquisition time of 1 minute. Spectra were processed using python and nmrGlue. Comparison of data obtained with Seedless and rectangular pulses are presented (**Supplementary Fig. 3**).

S.3.3 Water suppressed 1D perfect echo 1D

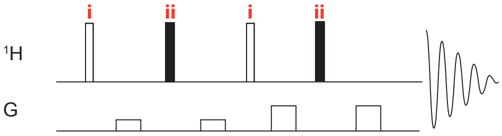

A Seedless suppressed perfect echo pulse sequence was created by taking a perfect-echo 1D sequence and substituting the pulses for Seedless pulses. For a water sample, the 180° pulses (24.5 kHz) are flanked by selective water 90° pulses (201 Hz). The suppressed spectra (**Main Text Fig. 2a**) were recorded with 9,761 complex points and a sweep width of 7,183Hz.

The interscan delay was 1s and 64 transients were recorded per FID for a total acquisition time of 6 minutes.

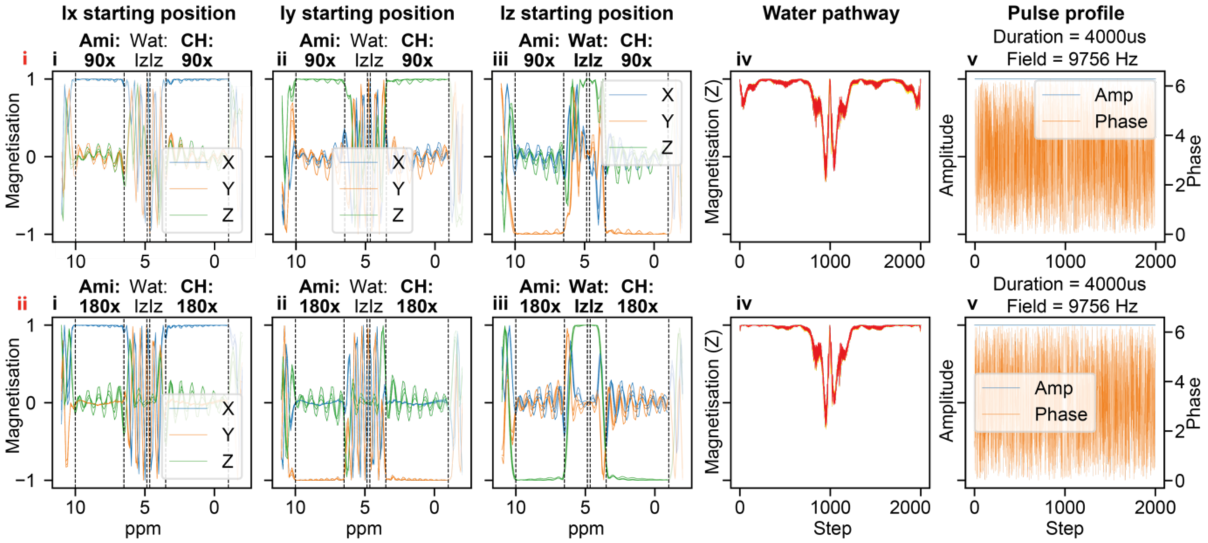

The suppression pulses were designed to excite the aliphatic (CH, -1 to 3.5 ppm, 15 frequencies) and the amide (NH, 6.5 ppm to 1ppm, 20 frequencies) regions, while applying the 'miss' restraint to water (4.65 to 4.85 ppm, 10 frequencies). Best results were achieved with unitary pulses, where 90° (**A**) and 180° (**B**) rotations are required. To monitor the success of the 'miss' process, the evolution progress of the water band is monitored (iv). Achieving perfect avoidance of water with these restraints is not possible and so the results represent a compromise. When compared to the excitation profiles of other pulses in this work, the compromise is visually apparent. The field for the pulses is lower than the maximum possible at 9.756 kHz. When operating at maximum B_1 in ^{1}H (25 kHz), water is highly excited rendering it impossible to restrain along a single trajectory. If the B_1 field is reduced too far, then it becomes impossible to simultaneously excite both the aliphatic and amide regions. Provided one can tolerate the loss of spins in the region 3.5 to 6.5 ppm (at 600 MHz) receiving no excitation, this pulse sequence will provide near perfect water suppression as shown in the text (**Main Text Fig. 2A**). The ring shifted methyl protons (S/N 4 in both spectra) allowed for an approximate mapping of water signal to an effective concentration, equal to 560 μM/7.5

μM in the rectangular and Seedless experiments respectively (with suppression factors of 10^5 and 10^7). Calculation of these pulses is considerably more demanding than all others in this work, as described in **Supplementary Note 2.6**. Owing to the added complexity, the 'miss' pulses took ca. 25 s for calculation (**Supplementary Fig. 3**).

S.3.4 Sensitivity enhanced ^{15}N HSQC

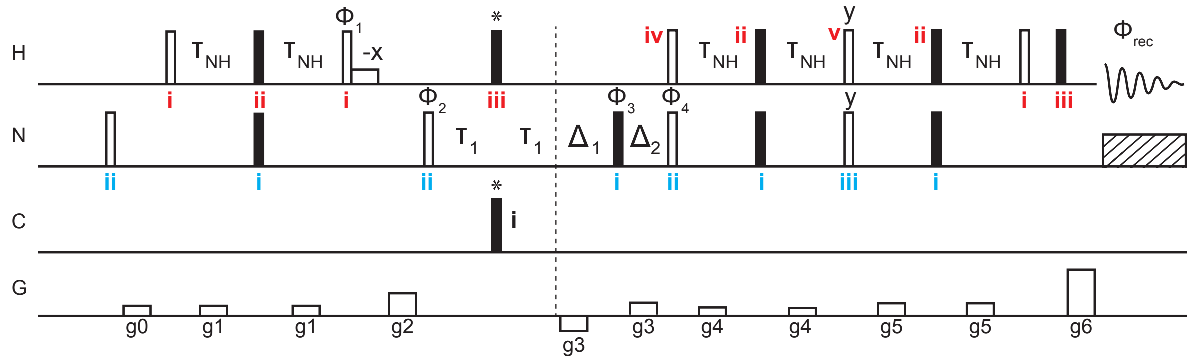

Data shown in **Main Text Fig. 2B** and **Supplementary Fig. 5**. The phase cycle is Φ_1 = 1, Φ_2 = 0, Φ_3 = (0,0,1,1,2,2,3,3), Φ_4 = 0 and Φ_{rec} = (0,0,2,2). ^{15}N indirect detection is achieved using the Rance-Kay method where 180° is added to Φ_4 and the sign of g6 are simultaneously altered for quadrature. The ratio of the product of the strengths and duration of g3 (applied in a bipolar fashion) and g6 are set to match the ratio of the ^{15}N and ^{1}H gyromagnetic ratios. For TPPI, 180° are added to Φ_2 and Φ_{rec} for every even numbered indirect time increment. The durations for gradients g0, g1, g2, g4 and g5 were set to 1 ms, 400 μs, 1.5 ms, 500 μs and 500 μs at 25%, 12.5%, 46.8%, 25% and 32.5% respectively to de-phase off-pathway magnetization. Δ_2 was set to Δ_1 plus the maximum duration of the two starred pulses.

600 MHz:

The 600Mhz 2D sensitivity enhanced NHSQC spectra with rectangular, and with Seedless pulses (**Main Text Fig. 2b**) were uniformly acquired with 50 (^{15}N) and 801 (^{1}H) complex points.

The sweep widths were set to 1,700 Hz (^{15}N) and 8,013 Hz (^{1}H). The interscan delay was 1 s with 4 transients per FID for a total duration of 9 minutes. The N-H INEPT was performed with τ_{NH}=0.0024 s. ^{15}N decoupling was achieved during the direct dimension using a waltz16 pattern at a field of 3,134 Hz. During the ^{13}C indirect acquisition, half of the dwell time was added to invert the sign of aliased peaks, requiring a 90°/180° zero/first order phase correction. The spectra were processed within the nmrPipe workflow embedded in the UnidecNMR GUI, with zero-filling and a sine-bell window function in both dimensions. All peaks were quantified using the UnidecNMR deconvolution algorithm and compared using python scripts.

950 MHz:

Recorded on a 950-MHz spectrometer with Bruker Avance III HD console and 5-mm TCI CryoProbe. The 950 MHz 2D sensitivity enhanced ^{15}N HSQC spectra with rectangular and Seedless pulses (**Main Text Fig. 2b**) were uniformly acquired with 128 (^{15}N) and 810 (^{1}H) complex points. The sweep widths were set to 3,849 Hz (^{15}N) and 11,433 Hz (^{1}H). The interscan delay was 1 s with 4 transients per FID for a total duration of 9 minutes. The ^{15}N decoupler during direct acquisition used the waltz65 decoupler pattern at a field of 1,968 Hz. The N-H INEPT was performed with τ_{NH}=0.00263 s. The spectra were processed within the nmrPipe workflow embedded in the UnidecNMR GUI, with zero-filling and a sine-bell window function in both dimensions. All peaks were quantified using the UnidecNMR deconvolution algorithm and compared using python scripts.

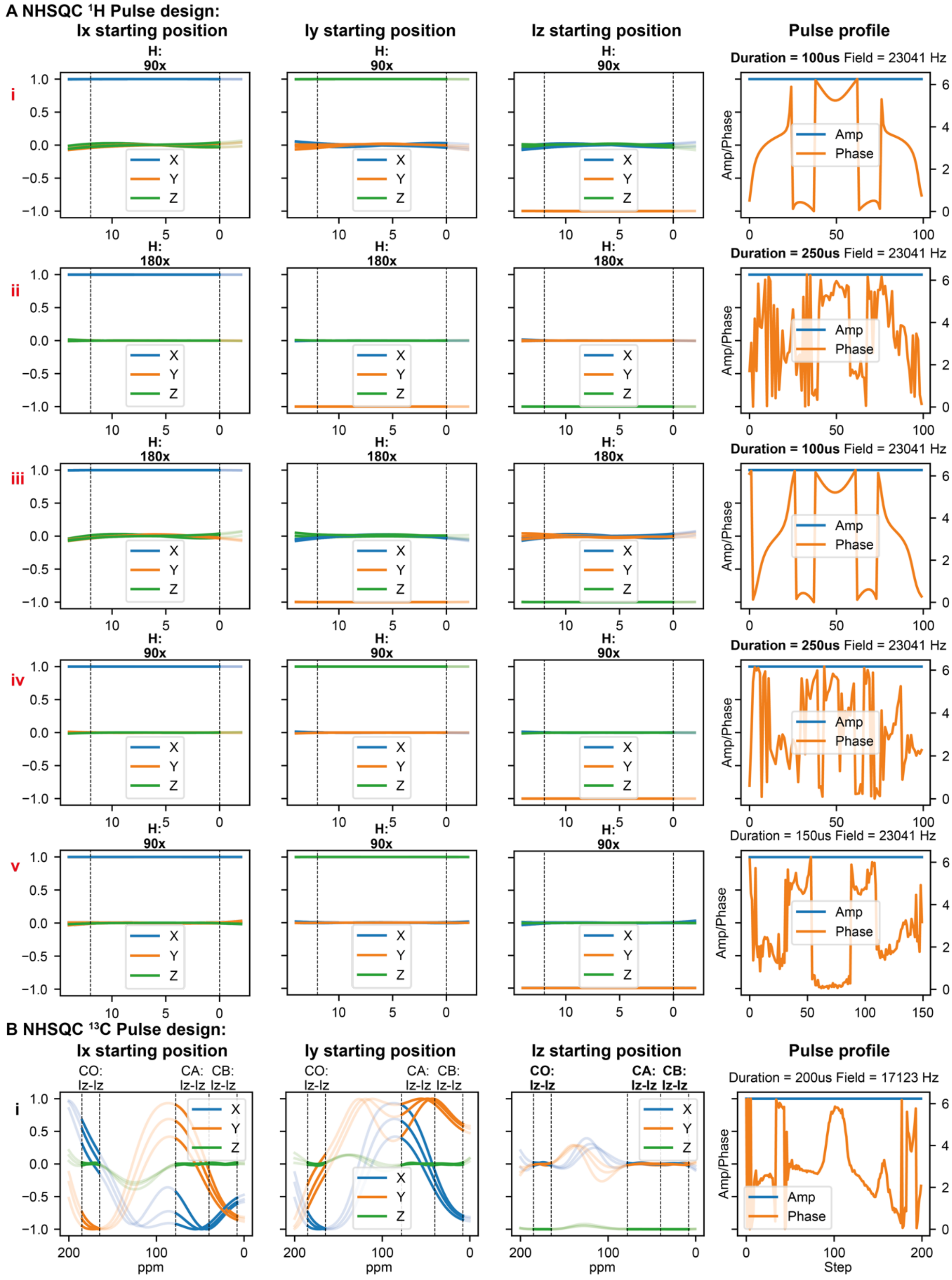

A NHSQC ¹H Pulse design:
Ix starting position
Iy starting position
Iz starting position
Pulse profile
i
ii
iii
iv
v
H: 90x
H: 180x
Duration = 100us Field = 23041 Hz
Duration = 250us Field = 23041 Hz
Duration = 150us Field = 23041 Hz
X
Y
Z
Amp
Phase
Amp/Phase
B NHSQC ¹³C Pulse design:
CO: Iz-Iz
CA: Iz-Iz
CB: Iz-Iz
Duration = 200us Field = 17123 Hz
ppm
Step

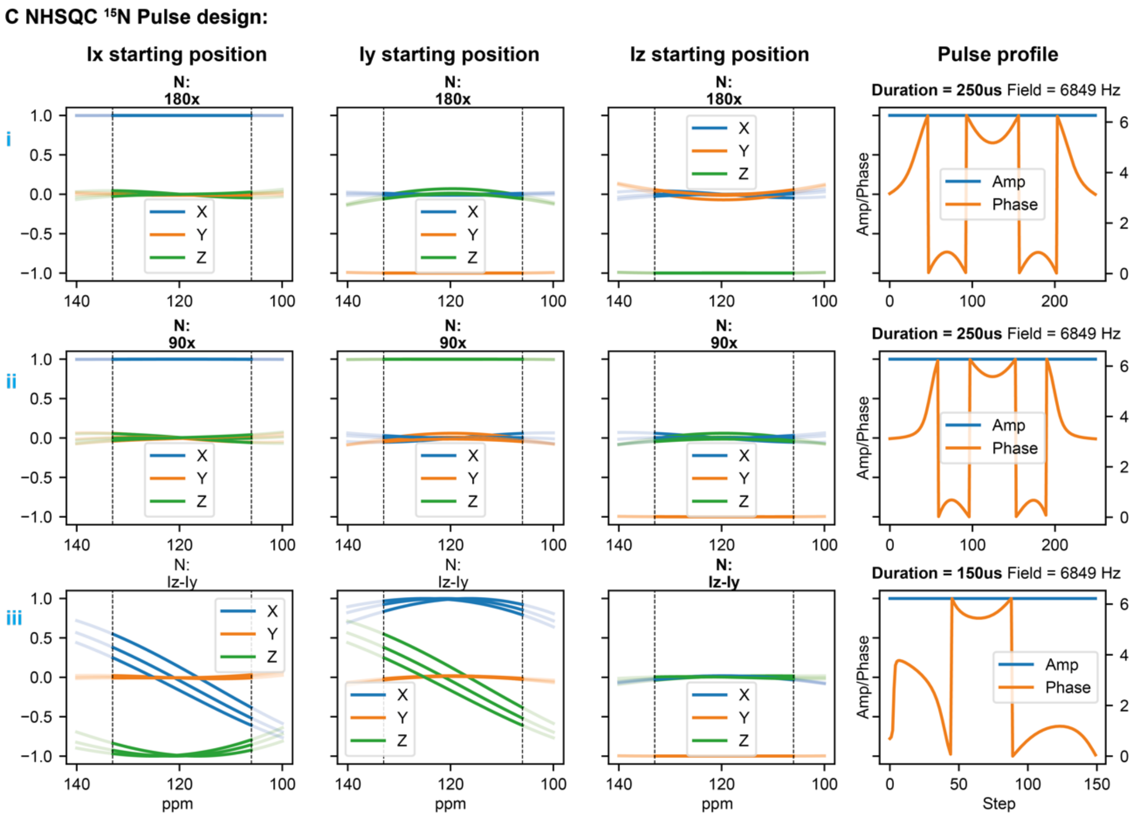

Four ^{1}H (A), one ^{13}C (B) and three ^{15}N (C) pulses were required to implement the HSQC using Seedless pulses. For ^{1}H and ^{15}N all 180° pulses are refocusing transverse magnetization spread out in the XY plane and so need to be unitary. For the decoupling ^{13}C 180° pulse (B), we need only to apply an inversion transform $\mathrm{Z} \rightarrow -\mathrm{Z}$, which can be accomplished with a state-to-state restraint. Where pulses are applied simultaneously, their durations are matched. The 90° ^{15}N pulses were unitary (Cii) when two magnetization pathways had to be handled, which is the case during the refocused INEPT where pulses have to simultaneously apply both $\mathrm{Z} \rightarrow -\mathrm{Y}$ and $\mathrm{X} \rightarrow -\mathrm{X}$ transforms. When only excitation/de-excitation is required, it is sufficient to apply state-to-state pulses performing $\mathrm{Z} \rightarrow -\mathrm{Y}$ which resulted in shorter pulses (150 μs versus 250 μs, C iii). We time matched the simultaneously applied ^{1}H 90-degree pulse (Av) to prevent unwanted evolutions.

At 600 MHz (**Main Text Fig. 2B**), all ^{1}H pulses were set to 100 μs total duration at 23.584 kHz (the maximum field possible, corresponding to a 10.4 μs duration 90° excitation), ^{15}N state-to-

state pulses were 150 μs and ^{15}N unitary pulses were 250 μs both at 7.042 kHz (corresponding to a 35.5 μs 90° excitation). The carbon refocusing during indirect acquisition was set to 200 μs at 17.605 kHz (corresponding to a 14.2 μs 90° excitation). At 950 MHz (**Main Text Fig. 2B**), all pulses were set to 250 μs for simplicity (including ^{15}N, ^{1}H and ^{13}C). The pulses were designed for maximum amplitude at the following fields: ^{1}H 17,421 kHz, ^{13}C 20 kHz and ^{15}N 7.225 kHz. The behaviour of both Seedless sequences is shown compared to the same sequence setup with rectangular pulses on both spectrometers (**Supplementary Fig. 5**).

S.3.5 Sensitivity enhanced HNCO

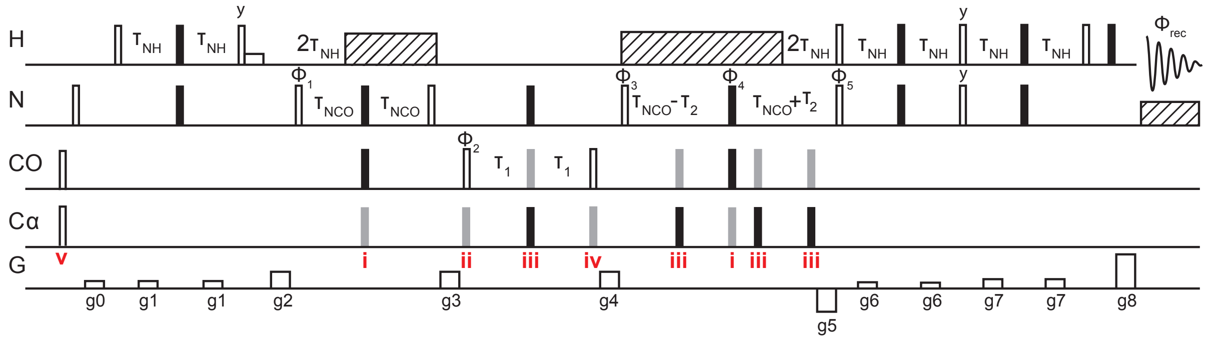

Data shown in **Main Text Figs. 3, 4** and **5**. The phase cycle is $\Phi_1 = 0$, $\Phi_2 = (0,2)$, $\Phi_3 = (0,0,2,2)$, $\Phi_4 = (0,2)$, $\Phi_5 = 0$ and $\Phi_{rec} = (0,2,2,0)$. Rance-Kay acquisition was employed for ^{15}N, achieved using gradients g5 and g8 (as per **Supplementary Note 3.4**) with quadrature achieved by adding 180° to Φ_5 and inverting the sign of g6. In ^{13}C, quadrature is achieved by adding 90° to Φ_2. For TPPI, the signs of Φ_2 and Φ_{rec} were inverted for even numbered indirect time increments for ^{13}C and Φ_3 and Φ_{rec} for ^{15}N indirect. The gradient durations g0, g1, g2, g3 and g4, g6 and g7 were set to 500 μs, 500 μs, 2 ms, 750 μs, 1 ms, 300 μs and 200 μs at 25%, 15.6%, 46.8%, 62.5%, 46.8%, 15.6% and 31.25% respectively to de-phase off-pathway magnetization.

The 3D sensitivity enhanced HNCO spectra (**Main Text Fig. 4a**) were acquired with random sampling of indirect dimensions. 25 (^{13}C) and 15 (^{15}N) complex points were randomly chosen from ranges of 0 to 49 and 0 to 29 respectively for 25% sparsity. The direct dimension was uniformly acquired with 800 (^{1}H) complex points. The sweep widths were set to 1,750 Hz (^{13}C), 1,600 Hz (^{15}N) and 7,994 Hz (^{1}H). The interscan delay was 1.2s and 4 transients per FID for a total duration of 2 hours 18 minutes. The ^{15}N decoupler during direct acquisition used the waltz16 decoupler pattern at a field of 3,508 Hz. The ^{1}H decoupler used the waltz16 decoupler pattern at a field of 7,812 Hz. The N-H and N-CO INEPTs were performed with τ_{NH}=0.0023 s and τ_{NCO}=0.0124 s delays, respectively. During the ^{13}C indirect acquisition, half of the dwell time was added to invert the sign of aliased peaks, requiring a 90°/180° zero/first order phase correction. The spectra were processed within the nmrPipe workflow embedded in the UnidecNMR GUI, with zero-filling and a sine-bell window function in all dimensions and indirect dimension reconstruction using the SMILE algorithm. All peaks were quantified using the UnidecNMR deconvolution algorithm and compared using python scripts.

The 2D sensitivity enhanced HNCO spectra (**Main Text Fig. 4**) were uniformly acquired with 50 (^{13}C) and 800 (^{1}H) complex points. The sweep widths were set to 1,750 Hz (^{13}C) and 7,994 Hz (^{1}H). The interscan delay was 1.2 s with 4 transients per FID for a total duration of 11 minutes. All other parameters were the same as the 3D counterparts. The spectra were processed within the nmrPipe workflow embedded in the UnidecNMR GUI, with zero-filling and a sine-bell window function in both dimensions. All peaks were quantified using the UnidecNMR deconvolution algorithm and compared using python scripts.

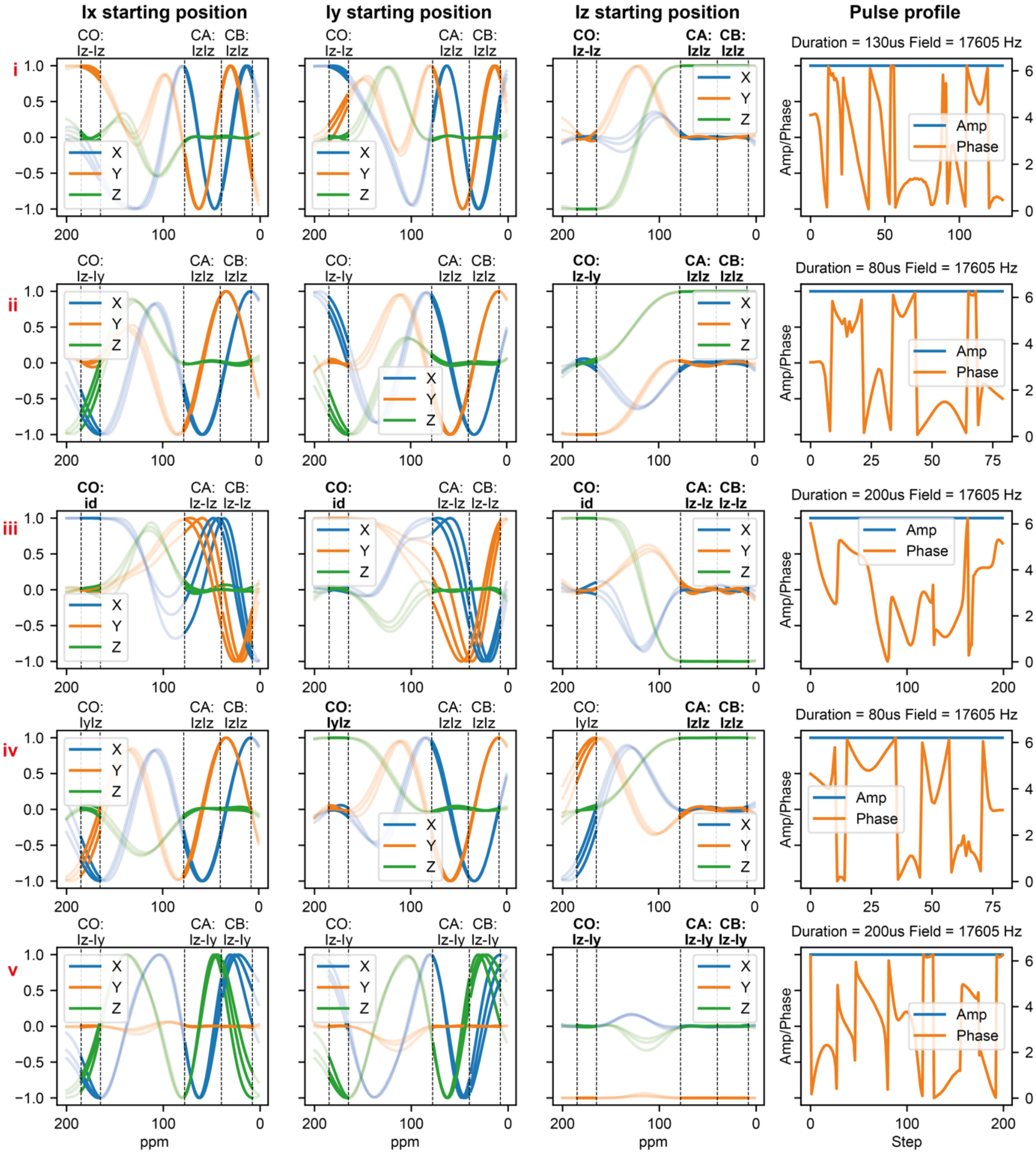

^{1}H and ^{15}N pulses were implemented as described for the ^{15}N HSQC (**Supplementary Note 3.4**). For ^{13}C, the carrier was set to CO (176 ppm) for all pulses. The initial ^{15}N and ^{13}C pulses (v) were state-to-state $\mathrm{Z} \rightarrow -\mathrm{Y}$, used to de-phase equilibrated heteronuclear magnetisation. The first INEPT block employs ^{1}H state-to-state $\mathrm{Z} \rightarrow -\mathrm{Y}$ excitation (100 μs) and $\mathrm{Y} \rightarrow \mathrm{Z}$ (100 μs) de-excitation pulses. The 180° for the active INEPT spin (^{1}H) must be a unitary 180°x pulse (250 μs), while the passive spin (^{15}N) can be a state-to-state $\mathrm{Z} \rightarrow -\mathrm{Z}$ inversion pulse (250 μs). The N → CO INEPT employs both $Nz \rightarrow -\mathrm{Ny}$ state-to-state excitation and $\mathrm{Ny} \rightarrow -\mathrm{Nz}$ state-to-

state de-excitation pulses with a unitary 180° pulse for ^{15}N and a $\mathrm{Z} \rightarrow -\mathrm{Z}$ state-to-state ^{13}C inversion pulse on CO, while performing a $\mathrm{Z} \rightarrow \mathrm{Z}$ state-to-state transform on Cα+Cβ to 'hold' them in place (pulse **i**). The 90° pulses prior to indirect acquisition of CO are state-to-state $\mathrm{Z} \rightarrow -\mathrm{Y}$ (pulse **ii**) and $\mathrm{Y} \rightarrow \mathrm{Z}$ for CO and $\mathrm{Z} \rightarrow \mathrm{Z}$ for Cα+Cβ (pulse **iv**).

During acquisition, decoupling of Cα/Cβ is accomplished (pulse **iii**) by inverting Cα/Cβ while performing the identity operation on CO. This has the result of leaving CO evolution uninterrupted during the decoupling of Cα/Cβ . The constant-time ^{15}N acquisition (CT) block begins with a $\mathrm{Z} \rightarrow -\mathrm{Y}$ transform and uses a unitary 180°x pulse on ^{15}N for refocusing, together with pulse **iii** to invert Cα+Cβ while holding CO in place, and pulse **i** which inverts CO while holding Cα+Cβ.

The sensitivity enhanced (SE) block uses the same approach as the sensitivity enhanced ^{15}N HSQC (**Supplementary Note 3.4**) with unitary 90° pulses on ^{15}N to handle both magnetization pathways, and the 180° pulses either being inversions for $\mathrm{Z} \rightarrow -\mathrm{Z}$ or 180° universal pulses as required. The pulses were designed with the same fields as before (**Supplementary Note 3.4**).

For completeness, we compared the sensitivity of the HNCO sequence to one from the bruker standard library, 'hncocacbgp3d', constructed using Q' shaped pulses (**Main Text Fig. 5**). The sequence is:

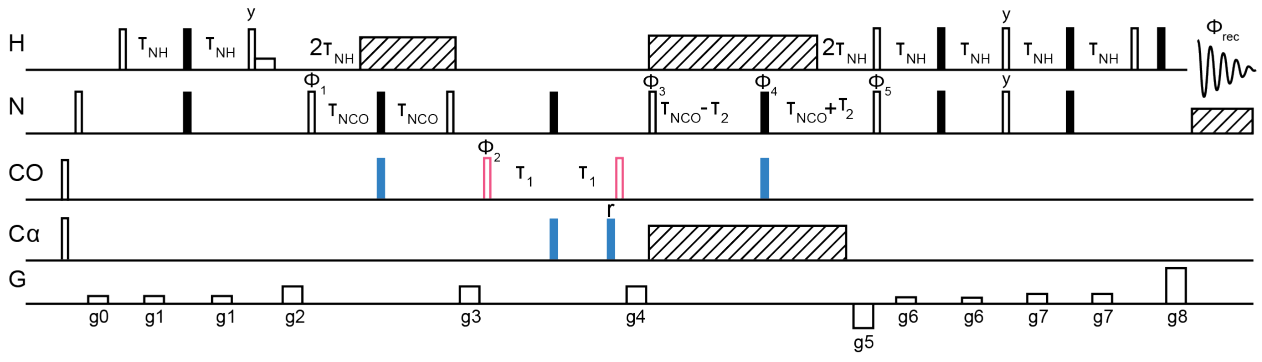

The pulses in red are Q5, applied for 320 μs at 14.4 kHz that give a 90° rotation. The blue pulses are Q3, applied for 256 us at 12.9 kHz. The indicator 'r' is a Q3 pulse that is time and

phase reversed, which is necessary to minimise phase errors associated with this pulse. Cα decoupling was achieved with a seduce pulse train applied at 847 Hz. The phase cycle is $\Phi_1 = 0$, $\Phi_2 = (0,2)$, $\Phi_3 = (0,0,2,2)$, $\Phi_4 = (0,2)$, $\Phi_5 = 0$ and $\Phi_{rec} = (0,2,2,0)$. Rance-Kay acquisition was employed for ^{15}N, achieved using gradients g5 and g8 (as per **Supplementary Note 3.4**) with quadrature achieved by adding 180° to Φ_5 and inverting the sign of g6. In ^{13}C, quadrature is achieved by adding 90° to Φ_2. For TPPI, the signs of Φ_2 and Φ_{rec} were inverted for even numbered indirect time increments for ^{13}C and Φ_3 and Φ_{rec} for ^{15}N indirect. The gradient durations g0, g1, g2, g3 and g4, g6 and g7 were set to 500 μs, 500 μs, 2 ms, 750 μs, 1 ms, 300 μs and 200 μs at 25%, 15.6%, 46.8%, 62.5%, 46.8%, 15.6% and 31.25% respectively to de-phase off-pathway magnetization. Acquisition parameters were identical to that used for the Seedless sequence as described above. When implementing a pulse sequence using Q pulses, care needs to be taken to account for evolution during the pulses, which is unnecessary when implementing seedless pulses.

S.3.6 Sensitivity enhanced HNCA

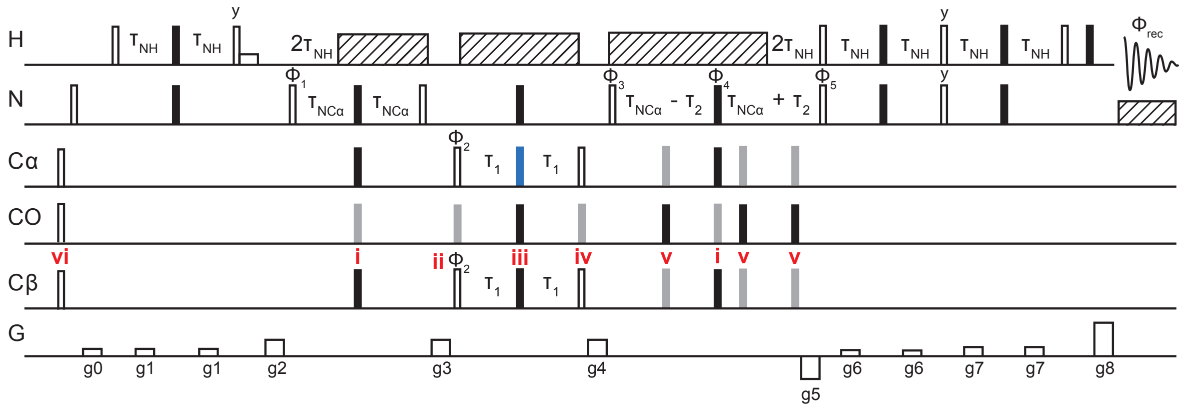

Shown in **Main Text Fig. 5**. The phase cycles are $\Phi_1 = (0,2)$, $\Phi_2 = (0,0,2,2)$, $\Phi_3 = 0$, $\Phi_4 = (0,0,0,0,2,2,2,2)$, $\Phi_5 = 0$ and $\Phi_{rec} = (0,2,2,0)$. Rance-Kay acquisition was achieved for ^{15}N using g5 and g8, with 180° being added to Φ_5 and the sign of g6 being inverted for quadrature detection. Quadrature detection for ^{13}C was achieved by adding 90° to Φ_2. For TPPI, Φ_2 and Φ_{rec} were inverted for even increments for ^{13}C and Φ_3 and Φ_{rec} for ^{15}N. Gradients g0, g1, g2, g3 and g4, g6 and g7 were set to 500 μs, 500 μs, 1 ms, 500 μs, 600 μs, 500 μs and 300 μs at

25%, 12.5%, 31.25%, 15.625%, 31.25%, 25% and 6.25% respectively to de-phase off-pathway magnetization.

3D sensitivity enhanced HNCA spectra (**Main Text Fig. 4b**) were acquired with random sampling of indirect dimensions. 14 (^{13}C) and 15 (^{15}N) complex points were randomly chosen from ranges of 0 to 49 and 0 to 29 respectively for 14% sparsity. The direct dimension was uniformly acquired with 899 (^{1}H) complex points. The sweep widths were set to 2,250 Hz (^{13}C), 1,210 Hz (^{15}N) and 8,993 Hz (^{1}H). The interscan delay was 1.2 s with 16 transients per FID for a total duration of 5 hours 9 minutes. The ^{15}N decoupler during direct acquisition used the waltz16 decoupler pattern at a field of 3,030 Hz. The ^{1}H decoupler used the waltz16 decoupler pattern at a field of 8,176 Hz. The N-H and N-Ca INEPTs were performed with τ_{NH}=0.0023s and τ_{NCa}=0.0124s delays, respectively. During the ^{13}C indirect acquisition, half of the dwell time was added to invert the sign of aliased peaks, requiring a 90°/180° zero/first order phase correction. The spectra were processed within the nmrPipe workflow embedded in the UnidecNMR GUI, with zero-filling and a sine-bell window function in all dimensions and indirect dimension reconstruction using the SMILE algorithm. All peaks were quantified using the UnidecNMR deconvolution algorithm and compared using python scripts.

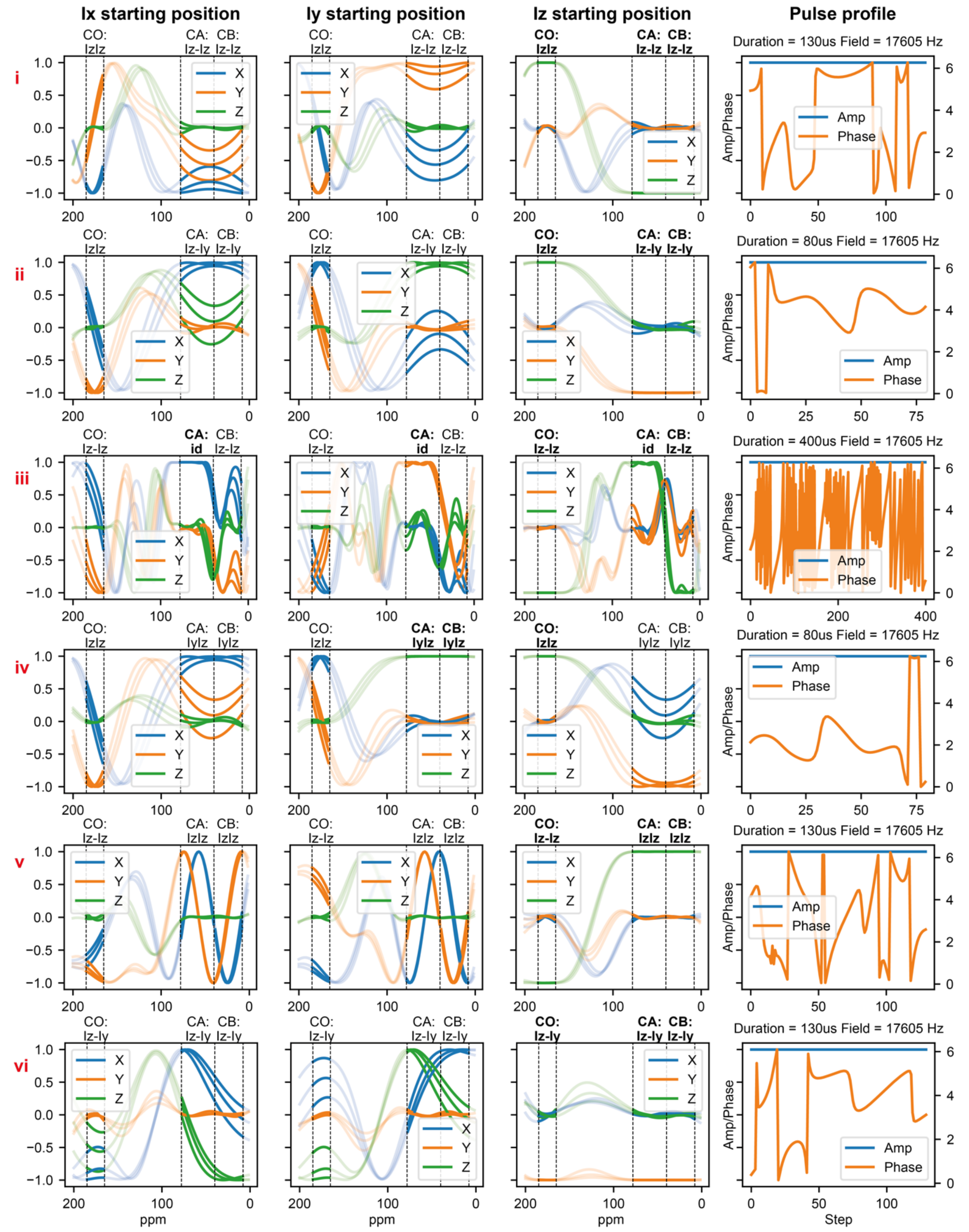

^{1}H and ^{15}N pulses were designed as described for the ^{15}N HSQC (**Supplementary Note 2.3.4**) and only ^{13}C pulses are shown. The carrier for all pulses was set to 58 ppm, the centre of the CA band. The first nitrogen and carbon (pulse **vi**) pulses are state-to-state $Z \rightarrow -Y$, used to eliminate equilibrate heteronuclear magnetisation. The first INEPT block employs ^{1}H state-to-

state 90-degree $\mathrm{Z} \rightarrow -\mathrm{Y}$ excitation (100 µs) and $\mathrm{Y} \rightarrow \mathrm{Z}$ (100 µs) de-excitation pulses. During the first INEPT, the 180° for the transverse spin (^{1}H) must be a unitary (250 us), while the passive spin (^{15}N) can be a $\mathrm{Z} \rightarrow -\mathrm{Z}$ inversion pulse (250 µs). The second N → Cα INEPT employs $Nz \rightarrow -\mathrm{Ny}$ state-to-state excitation and $Ny \rightarrow \mathrm{Nz}$ de-excitation pulses. For transfer, a unitary 180°x was required for the transverse spin (^{15}N) while for ^{13}C a $\mathrm{Z} \rightarrow -\mathrm{Z}$ inversion pulse on Cα+Cβ enables N-Cα transfer, while a state-to-state $\mathrm{Z} \rightarrow \mathrm{Z}$ restraint on CO (pulse **i**) decouples this nucleus from the transfer. While our desired transfer is N → Cα, the typical coupling constant between N and Cβ is comparatively minimal and therefore no further Cα/Cβ selectivity is required.

The 90-degree pulses for indirect acquisition of ^{13}Cα are state-to-state 90° $\mathrm{Z} \rightarrow -\mathrm{Y}$ (pulse **ii**) and $\mathrm{Y} \rightarrow \mathrm{Z}$ for Cα+Cβ (pulse **iv**) and $\mathrm{Z} \rightarrow \mathrm{Z}$ for CO. The most demanding pulse in the sequence (pulse **iii**) performs an identity operation on Cα together with a $\mathrm{Z} \rightarrow -\mathrm{Z}$ inversion on CO and Cβ. This effectively decouples Cα from these two nuclei, without interrupting its chemical shift evolution. The longer duration (400 µs) is necessary to get a sharp transition between the chosen Cα/Cβ interval. The constant-time Nitrogen acquisition (CT) block, and the sensitivity enhanced refocusing block follow considerations identical to the HNCO (**Supplementary Note 3.6**).

S.3.7 Sensitivity enhanced HNCACO

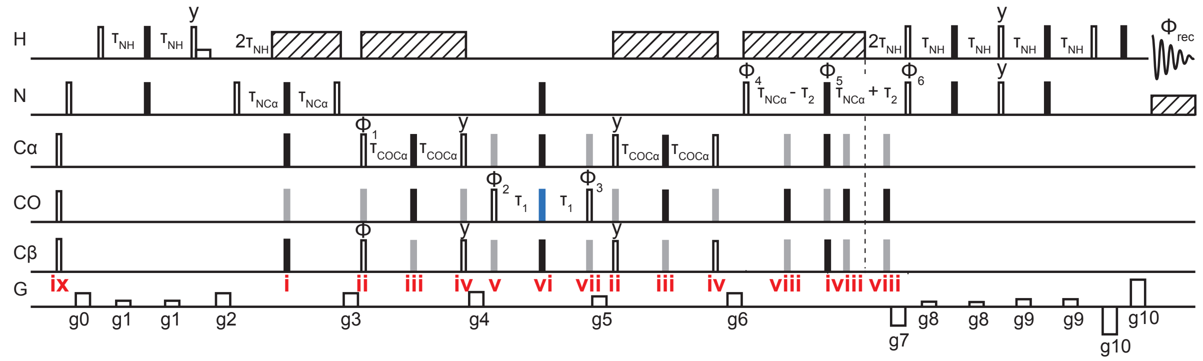

Shown in **Main Text Figs 3 and 5**. The phase cycles were Φ_1 = (0,2), Φ_2 = (0,0,2,2), Φ_3 = 0, Φ_4 = 0, Φ_5 = (0,0,0,0,2,2,2,2), Φ_6 = 0 and Φ_{rec} = (0,2,2,0). For Rance-Kay acquisition in ^{15}N,

the strengths and durations of g7 and g10 (which is bipolar) were matched appropriately with 180° being added to Φ_6 and the sign of g7 being inverted for quadrature. Quadrature in ^{13}C was achieved by adding 90° to Φ_2. For TPPI, for even numbered time increments 180° was added to Φ_2 and Φ_{rec} for ^{13}C acquisition and Φ_4 and Φ_{rec} for ^{15}N. Gradients g0, g1, g2, g3 and g4, g5, g6, g8 and g9 were set to 500 μs, 500 μs, 1 ms, 1 ms, 1 ms, 1 ms, 600 μs, 500 μs and 300 μs at 25%, 12.5%, 31.25%, 3.125%, 46.875%, 25%, 31.25%, 15.625%, 6.25% respectively to de-phase off-pathway magnetization.

3D sensitivity enhanced HNCACO spectra (**Main Text Figs 3b, 5c**) were acquired with random sampling of indirect dimensions. 30 (^{13}C) and 12 (^{15}N) complex points were randomly chosen from ranges of 0 to 75 and 0 to 29 respectively for 16.6% sparsity. The direct dimension was uniformly acquired with 899 (^{1}H) complex points. The sweep widths were set to 1800 Hz (^{13}C), 1,620 Hz (^{15}N) and 8,993 Hz (^{1}H). The interscan delay was 1.2s and 16 transients per FID for a total duration of 9 hours 1 minute. During direct acquisition the ^{15}N decoupler used the waltz16 decoupler pattern at a field of 3,508 Hz. The ^{1}H decoupler used the waltz16 decoupler pattern at a field of 7,812 Hz. The N-H, N-Ca and Ca-CO INEPTs were performed with τ_{NH}=0.0023s, τ_{NCa}=0.0124s and τ_{CaCO}=0.0036s delays respectively. During the ^{13}C indirect acquisition, half of the dwell time was added to invert the sign of aliased peaks, requiring a 90°/180° zero/first order phase correction. The spectra were processed within the nmrPipe workflow embedded in the UnidecNMR GUI, with zero-filling and a sine-bell window function in all dimensions and indirect dimension reconstruction using the SMILE algorithm. All peaks were quantified using the UnidecNMR deconvolution algorithm and compared using python scripts.

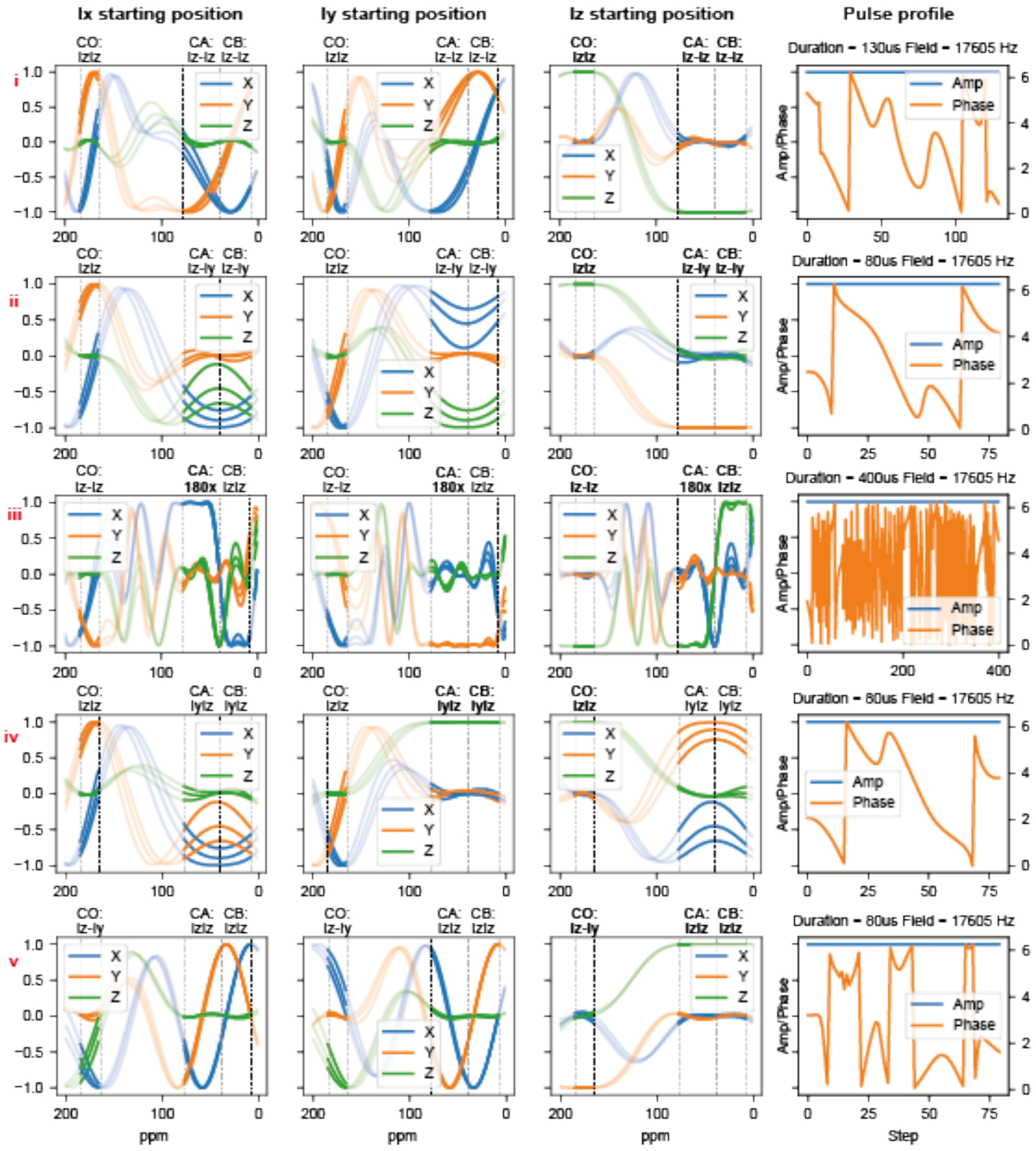
Ix starting position
Iy starting position
Iz starting position
Pulse profile
i
ii
iii
iv
v
CO:
CA:
CB:
Duration = 130us Field = 17605 Hz
Duration = 80us Field = 17605 Hz
Duration = 400us Field = 17605 Hz
Amp
Phase
Amp/Phase
X
Y
Z
ppm
Step

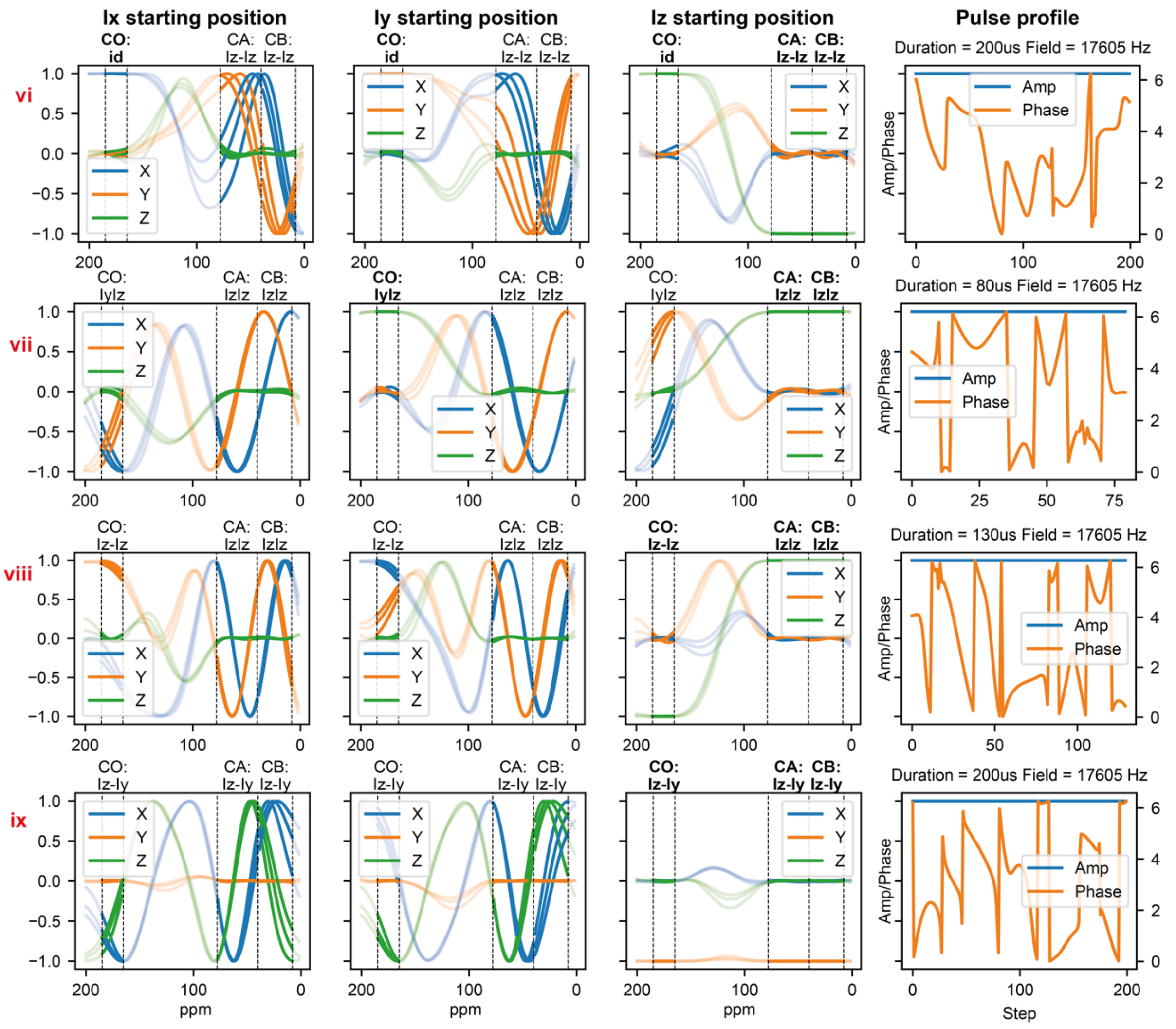

[1]H and ^{15}N pulses were set as already described (**Supplementary Note 3.4**). Nine 13C pulses were required, with the carrier set to CO (176 ppm). The first ^{15}N and [13]C (pulse **ix**) pulses are state-to-state $\mathrm{Z} \rightarrow -\mathrm{Y}$ and are used to eliminate equilibrated heteronuclear magnetisation. The first INEPT block employs ^{1}H state-to-state $\mathrm{Z} \rightarrow -\mathrm{Y}$ excitation (100 μs) and $\mathrm{Y} \rightarrow \mathrm{Z}$ (100 μs) de-excitation pulses. During the first INEPT, the 180° pulse on the transverse spin (^{1}H) must be a unitary (250 μs) but can be a $\mathrm{Z} \rightarrow -\mathrm{Z}$ inversion pulse for the longitudinal spin (^{15}N, duration 250 μs).

The N → Cα INEPT employs $\mathrm{Nz} \rightarrow -\mathrm{Ny}$ state-to-state excitation and $\mathrm{Ny} \rightarrow \mathrm{Nz}$ de-excitation. A unitary 180°x was used for refocusing on ^{15}N, while the ^{13}C is a $\mathrm{Z} \rightarrow -\mathrm{Z}$ inversion pulse on Cα+Cβ to enable transfer, and $\mathrm{Z} \rightarrow \mathrm{Z}$ state-to-state on CO (pulse **i**) for decoupling. While our

desired transfer is N → Cα, the typical coupling constant between N and Cβ is small and therefore no further band selectivity is required.

The Cα → CO INEPT is more complex and employs a state-to-state $C\alpha_z \rightarrow -C\alpha_y$ excitation (pulse **ii**) and $C\alpha_y \rightarrow C\alpha_z$ de-excitation (pulse **iv**) (80 μs each). As we have transverse Cα magnetization, significant transfer to Cβ can occur without Cα/Cβ band selectivity. To prevent this, we need a pulse that performs a unitary 180° rotation of Cα, inverts CO to enable the INEPT transfer, but holds Cβ in place using a state-to-state $\mathrm{Z} \rightarrow \mathrm{Z}$ transform (400 μs, pulse **iii**).

For indirect acquisition of ^{13}CO, state-to-state 90° $\mathrm{Z} \rightarrow -\mathrm{Y}$ (pulse **v**) and $\mathrm{Y} \rightarrow \mathrm{Z}$ (pulse **vii**) for CO with $\mathrm{Z} \rightarrow \mathrm{Z}$ for Cα+Cβ were used. To decouple Cα/Cβ while not interrupting the evolution of CO requires a pulse that performs the identity on CO and inversion on Cα+Cβ (pulse **vi**). The return CO → Cα INEPT employs the same pulses as for the Cα → CO INEPT, where Cα/Cβ is crucial to avoid loss of signal. Similarly, the constant-time ^{15}N block and sensitivity enhanced refocused INEPT use the approaches already discussed for the HNCO (**Supplementary Note 3.5**, pulses **i** and **viii**)

S.3.8 Sensitivity enhanced HNCOCA

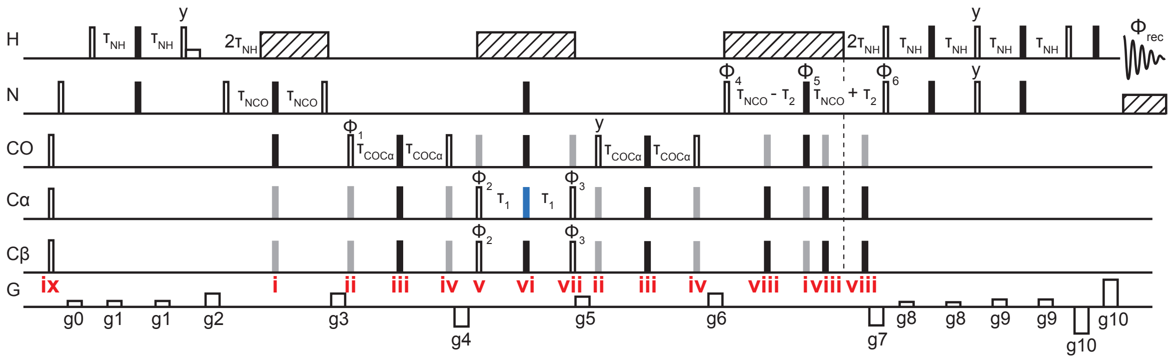

Shown in **Main Text Fig. 4**. The phase cycle was Φ_1 = (0,2), Φ_2 = (0,0,2,2), Φ_3 = 0, Φ_4 = 0, Φ_5 = (0,0,0,0,2,2,2,2), Φ_6 = 0 and Φ_{rec} = (0,2,2,0). Rance-Kay indirect acquisition was achieved

for ^{15}N where the strengths and durations of gradients g7 and g10 (bipolar) were matched as described for the ^{15}N HSQC (**Supplementary Note 3.4**) and 180° was added to Φ_6 together with inverting the sign of g7 to achieve quadrature. To achieve quadrature in ^{13}C, 90° were added to Φ_2. For TPPI, for even numbered time increments 180° was added to Φ_2 and Φ_{rec} for ^{13}C and Φ_4 and Φ_{rec} for ^{15}N. Gradients g0, g1, g2, g3 and g4, g5, g6, g8 and g9 were set to 500 μs, 500 μs, 1 ms, 1 ms, 1 ms, 1 ms, 600 μs, 500 μs and 300 μs at 25%, 12.5%, 31.25%, 3.125%, -46.875%, 25%, 25%, 15.625%, 6.25% respectively to de-phase off-pathway magnetization.

3D sensitivity enhanced HNCOCA spectra (**Main Text Fig. 4d**) were acquired with random sampling of indirect dimensions. 14 (^{13}C) and 15 (^{15}N) complex points were randomly chosen from ranges of 0 to 49 and 0 to 29 respectively for 14% sparsity. The direct dimension was uniformly acquired with 899 (^{1}H) complex points. The sweep widths were set to 2,250 Hz (^{13}C), 1,620 Hz (^{15}N) and 8,993 Hz (^{1}H). The interscan delay was 1.2s with 4 transients per FID for a total duration of 1 hours 19 minutes. During direct acquisition the ^{15}N decoupler used the waltz16 decoupler pattern at a field of 3,508 Hz. The ^{1}H decoupler used the waltz16 decoupler pattern at a field of 7812 Hz. The N-H, N-CO and N-Ca INEPTs were performed with τ_{NH}=0.0023s, τ_{NCO}=0.012s and τ_{COCa}=0.0041s delays respectively. During the ^{13}C indirect acquisition, half of the dwell time was added to invert the sign of aliased peaks, requiring a 90°/180° zero/first order phase correction. The spectra were processed within the nmrPipe workflow embedded in the UnidecNMR GUI, with zero-filling and a sine-bell window function in all dimensions and indirect dimension reconstruction using the SMILE algorithm. All peaks were quantified using the UnidecNMR deconvolution algorithm and compared using python scripts.

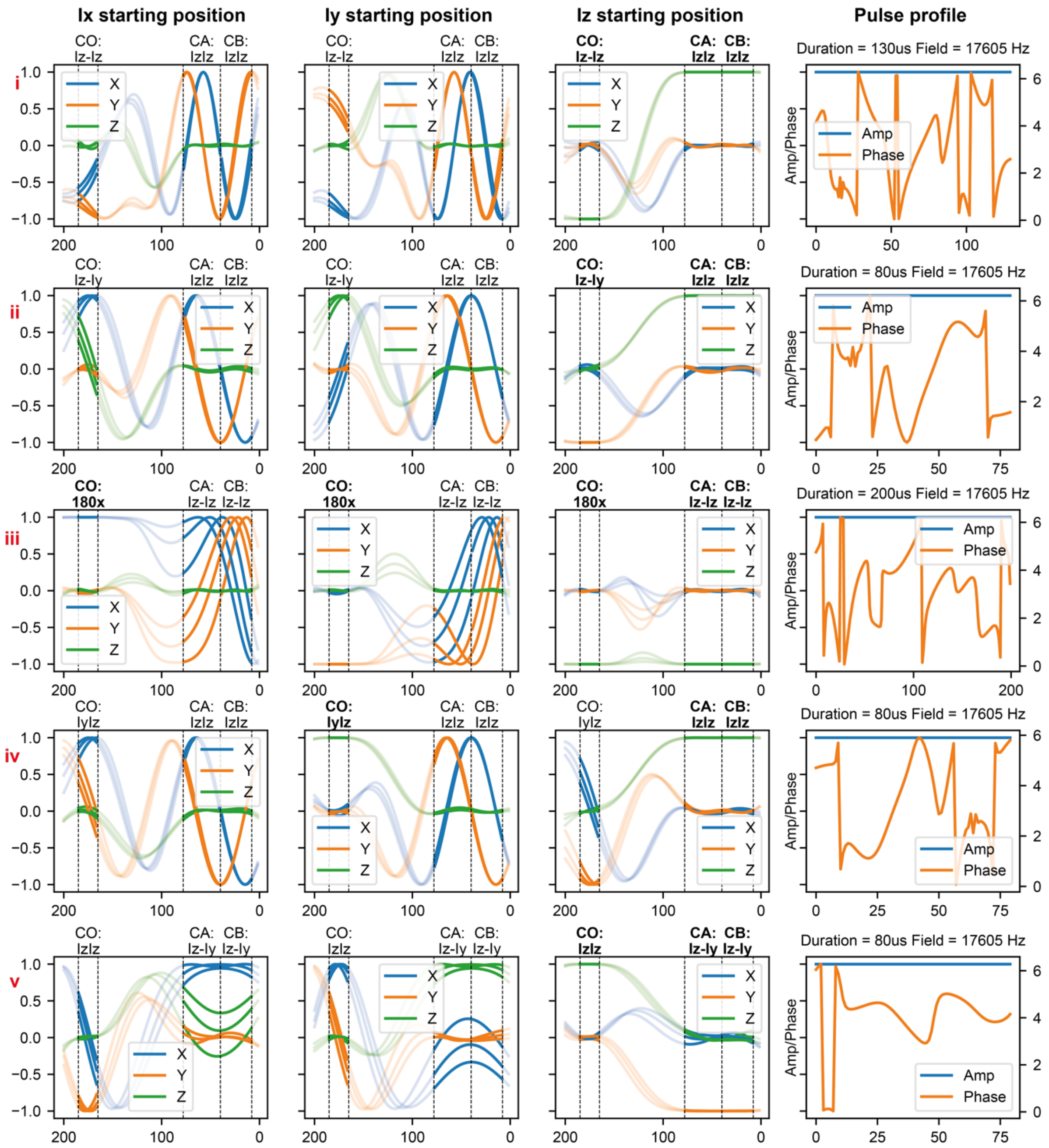

Ix starting position
Iy starting position
Iz starting position
Pulse profile
i
ii
iii
iv
v
CO: Iz-Iz
CA: IzIz
CB: IzIz
Duration = 130us Field = 17605 Hz
CO: Iz-Iy
Duration = 80us Field = 17605 Hz
CO: 180x
CA: Iz-Iz
CB: Iz-Iz
Duration = 200us Field = 17605 Hz
CO: IyIz
Duration = 80us Field = 17605 Hz
CO: IzIz
CA: Iz-Iy
CB: Iz-Iy
Duration = 80us Field = 17605 Hz
X
Y
Z
Amp
Phase
Amp/Phase

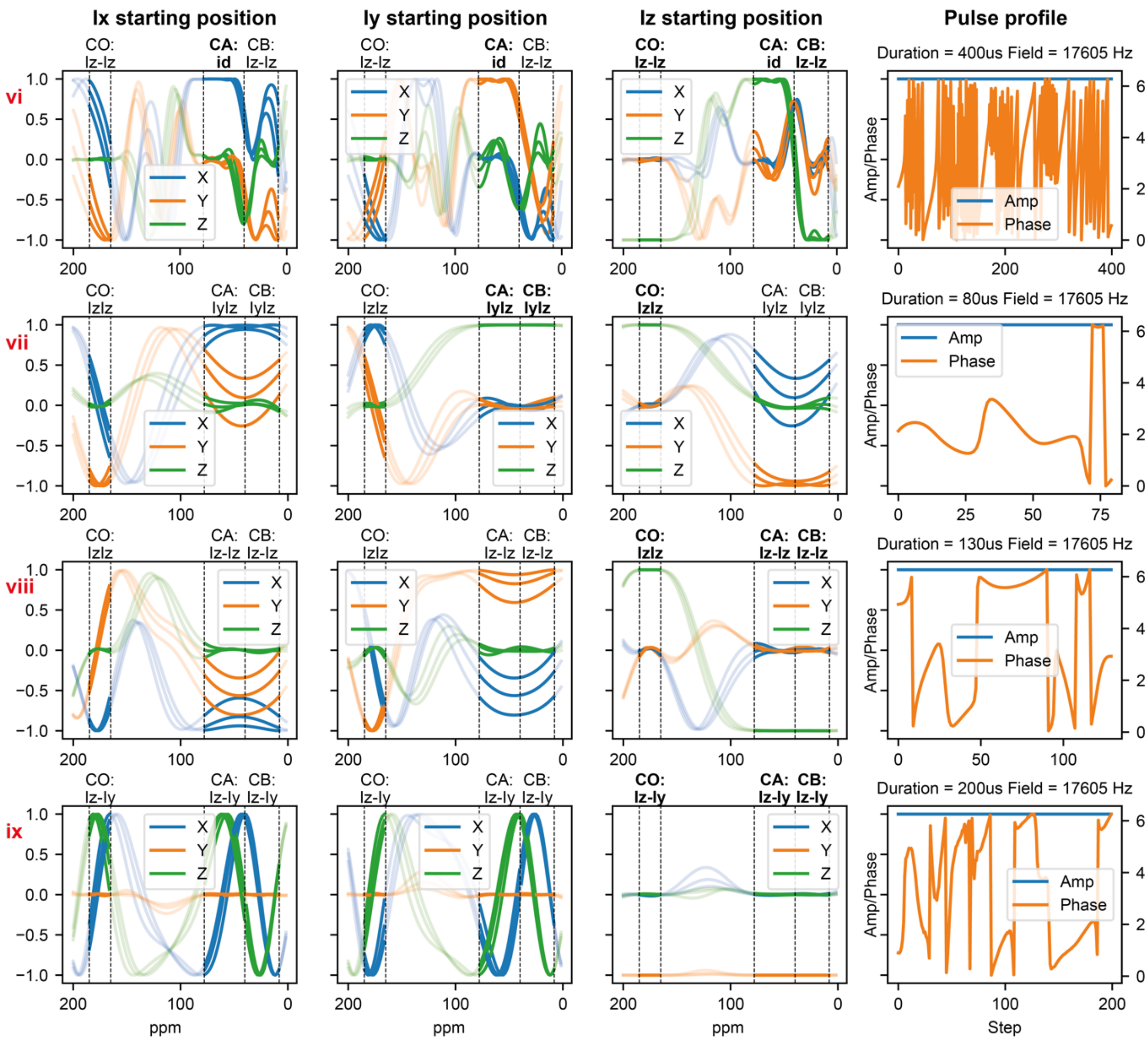

[1]H and ^{15}N pulses were as described earlier (**Supplementary Note 3.4**). Nine 13C pulses were required, centred on CA (58 ppm). The first ^{15}N and [13]C (**Bix**) pulses were unitary 90°x and were used to eliminate equilibrated heteronuclear magnetisation. The first INEPT block employs ^{1}H state-to-state $Z \rightarrow -Y$ excitation (100 μs) and $Y \rightarrow Z$ (100 μs) de-excitation pulses, a unitary 180° pulse for the transverse ^{1}H spins (250 μs), and a $Z \rightarrow Z$ inversion for [15]N (250 μs).

The N → CO INEPT employs $Nz \rightarrow -Ny$ state-to-state excitation and $Ny \rightarrow Nz$ de-excitation pulses, a unitary 180° pulse for the transverse 15N, a $Z \rightarrow -Z$ inversion on CO to ensure the transfer and a $Z \rightarrow Z$ transform on Cα+Cβ to decouple them (**Bi** 130 μs). The CO → Cα INEPT employs $CO_z \rightarrow -CO_y$ excitation (**Bii**) and $CO_y \rightarrow CO_z$ de-excitation pulses (**Biv**) (80 μs each)

with $Z \rightarrow Z$ on Cα+Cβ during both pulses. For the transfer, a unitary 180°C on CO is required together with a $Z \rightarrow -Z$ inversion on Cα+Cβ (**Biii**). As CO/Cβ coupling is negligible, Cα/Cβ selectivity is not required.

For indirect acquisition of Cα, we require state-to-state $Z \rightarrow -Y$ (**Bv**) and $Y \rightarrow Z$ (**Bvii**) excitation/de-excitation pulses on Cα/Cβ, and $Z \rightarrow Z$ on CO. No selectivity is required for Cα/Cβ as we do not generate coherent Cβ magnetization. To decouple CO and Cβ requires a more complex pulse that performs the identity on Cα, and $Z \rightarrow -Z$ on CO and Cβ (**Bvi**, 400 μs) as described for the HNCA (**Supplementary Note 3.6**).

The return Cα → CO INEPT employs the same pulses as the earlier CO → Cα transfer. The constant time ^{15}N and sensitivity enhanced refocused INEPTs were as described for the HNCO (**Supplementary Note 3.5**, pulses **Bviii**).

Supplementary Note 4

Seedless operation instructions

Scripts used to calculate all pulses in this manuscript are provided in the download, designed to be templates for new applications. The following input script found in Demo/HNCO/seedless.C generates the 5 ^{13}C pulses needed for the HNCO (**Supplementary Note 3.5**)

```
ncpus 8        # cpu count
seed 123       # Used to initialise pulses
frq150. (MHz)  # frequency of nucleus of interest
LINK           # pastes the durations/points and target lists together

outPath ./HNCO/C/  #out folder, end with a slash/ #make it first!
IterShow   50      #print output to screen every XXX
maxIter 5000       #maximum number of iterations

MakeSummary        #generate the seedless report.

# writeBruker #write bruker pulse file
writeVarian #write varian pulse file

RF: (B1/weight)   #one row per B1 homogeneity (1.0=perfect)
 0.93   0.25
 1.0    0.5
 1.05   0.25

Plot: (Min/Max/Num)  #for showing final profiles
 P 0  200   120      # will plot from 0 to 200 ppm, with 120 points.

SpinSystem: (Min/Max/Num/Target) #one row per group of spins
 CO  165  185  96    #setup the CO band: 96 spins between 165-185 ppm
 CA  40  78  96      #setup the CA band: 96 spins between 40-78 ppm
 CB  8  40  96       #setup the CB band: 96 spins between 8-40 ppm


Carriers: (ppm)
 176

wmH: (Hz)
17605 # 14.2us 90 time

Durations: (Total, points)
80E-6 80
80E-6 80
130E-6 130
200E-6 200
200E-6 200

Targets: (Wat Ali Ami)
 Iz -Iy , Iz Iz , Iz Iz   # Excitation of CO, hold CA/CB
 Iy Iz , Iz Iz , Iz Iz    # De-excitation of CO, hold CA/CB
 Iz -Iz , Iz Iz , Iz Iz   # CO inversion, hold CACB (N-CO INEPT)
 id , Iz -Iz , Iz -Iz     # pause CO evolution, CACB decouple
 Iz -Iy , Iz -Iy , Iz -Iy # Excite CO/CA/CB, used for initial purge
```

In more detail, the following commands can be set in the preamble of the script:

PAR	Default	Description
frq	150	Larmor frequency of nucleus (MHz)
outPath	./	Path for outputs.
writeVarian	false	Write a Varian pulse output file (true/false)
writeBruker	false	Write a Bruker pulse output file (true/false)
BrukerY	false	Treat Y as -Y
MakeSummary	false	Write full reports (needs gnuplot and latex to be in system path)
LINK	false	If included, the points/durations and target lists will be pasted.
seed	0	Seed for random number initial pulse creation
initPha	rand	Equal to 'rand' or 'const' for initial phases.
**Optimiser**		
gtol	0.9	Parameter to control the line search routine in libBFGS
maxIter	0	Set maximum number of iterations before convergence (0 means no maximum is set, and calculation halts only on convergence).
epsilon	1E-10	For convergence test in libBFGS.
ncpus	1	Number of cpus.
IterShow	100	Number of iterations that pass before printing progress to screen (set high for fast)
IterPrint	500	Number of iterations before creating output file (set high for fast)
NoCalcFid		If present, skip initial fidelity calculation/report prior to optimization (fast).
missWeight	100	Relative weighting for the 'miss' restraint applied to the suppression band.
GradStride	1	When applying 'miss' restraint, frequency of application (1 means every element, 2 means every other element).
**Read in**		
NoOpt		If present, do not run optimization (for simulation only)
Read		If reading in a pulse, for initialization / simulation, syntax presented below.
ReadMaxAmp	1023	Number for peak field in amplitudes (1023 for Varian, 100 for Bruker)
ReadColAmp	2	Column number in input file for amplitudes (1 for Bruker, 2 for varian)
ReadColPhi	1	Column number in input file for phase (2 for Bruker 1 for Varian)
**Amplitudes**		
OptAmp		If present, will optimize amplitudes.
alphaS	1E-6	Used as a smoothing function when optimizing amplitudes.
alpha	10	Used as a penalty when using amplitudes

Following the preamble, several sections can be specified. This includes a list of named frequency bands ("SpinSystem:"), and the B_1 inhomogeneity values to be considered ("RF:"), as well as the frequency range (in ppm) and number of frequencies used for the final plot ("Plot: "). Then lists are provided for the pulses. Each section can have one or more entries. These include Carriers (in ppm), peak field (in Hz), duration (total time and, number of required points, 'missweights', the factor that adjusts the weighting of the fidelity for the target restraints and the suppression restraints, and then the targets. The target row is comma "," delimited, and the number of targets must match the number of bands described above. Each entry can be set to one of:

- `90x`, `180x` or `id` for unitary rotations
- `Ia Ib` where a and b can be `X,Y,Z` or `-X, -Y, -Z` for state to state transforms

- `Iex` for XYcite.

Examples include, for ^{13}C where we have Cα, Cβ, CO bands (the number of restraints, separated by a comma, must match the number of frequency bands):

```
Iz Iz , Iz Iz , Iz -Iy
```

State-to-state z to z on Cα, z to z on Cβ, and z to -y on CO or

```
id , id , 180x
```

Identity on Cα, identity on Cβ, and a 180°x unitary rotation on CO.

A suppression restraint can be specified as:

```
Miss: (Min/Max/Num/Target) #one row per group of spins
 Wat  4.65  4.85  10  Iz Iz
```

If the indicator LINK is specified, then the list that describes the points/durations is pasted to the list that describes the targets. This is an efficient way to make a set of pulses with variable numbers of points and durations. By default, if N_C is the number of carriers specified, N_P is the number of points/durations specified, N_w is the number of fields and N_T is the number of targets, and N_M is the number of miss restraints, the code will produce $N_C.N_P.N_w.N_T.N_M$ total pulses. If LINK is specified, the code will produce $N_C.N_P.N_w.N_M$ pulses. Examples of the different cases are provided in the demonstration files.

If MakeSummary is specified, a report of the following type will be produced (shown for one of the ^{13}C pulses from the HNCO). The parameters of the pulse are detailed along with progress at various stages of the optimization. The program requires gnuplot and pdflatex to be in the system path to create the report. Inspection of the output shows probably too many iterations were specified, but as the computation took a second this isn't too much of a problem. Then we can see that magnetisation that starts on Z, for the CO is excited, and for Cα/Cβ it is held

as specified. But in the simulations for magnetisation that started on X or Y, the outcome is not well controlled. This is typical for a state-to-state pulse, where only the pathway specified ends up being controlled (here, Z), and the others are not. If the restraints were replaced with a 90x on CO, and the identity operation on Cα and Cβ, then we would see the expected control for magnestisation initially on X, Y and Z. Pulse optimisation will always be a compromise and the required level of control should match what is required in the sequence (see **methods**).

Seedless Report

Wednesday 27th November, 2024, 10:22

`CO_Iz-Iy_CA_IzIz_CB_IzIz_176_17605_80_100`

Spectrometer frequency:	150.00 MHz
Carrier:	176.00 ppm
ω_H:	17605.00 Hz
Equivalent 90° pulse:	14.20 μs
N:	80
dt:	1.00 μs
Duration:	80.00 μs

Region	min(ppm)	max(ppm)	spins	Condition
CO	165.00	185.00	96	Iz → -Iy
CA	40.00	78.00	96	Iz → Iz
CB	8.00	40.00	96	Iz → Iz

Relative B1	Weight
0.93	0.25
1.00	0.50
1.05	0.25

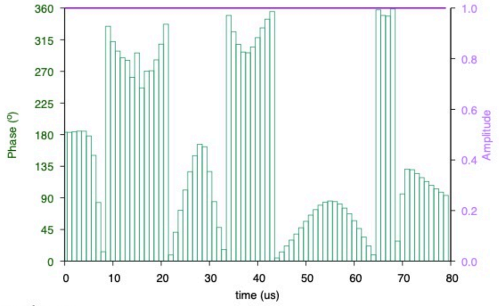

Performance
Infid: 6.6233e-04

Rotations

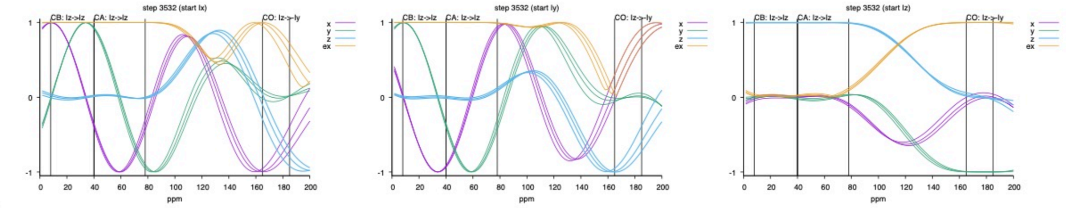

Convergence

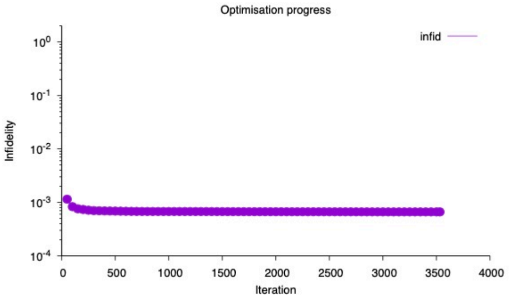

Optimiser settings
Amplitude initialisation: 1
Phase initialisation: rand
gtol: 0.90
maxIter: 5000
epsilon: 1.00e-10
Plot settings
Start operators: Ix Iy Iz
Min: 0.00 ppm Max: 200.00 ppm Points: 120 pskipMin: 0.00 pskipMax: 0.00

Supplementary Note 5.

Pulse performance comparisons

The current paradigm is for a user to take pre-calculated pulses, then rescale the power/duration to correctly match the specific spectrometer/sample, noting that increasing the duration by a factor will compress the bandwidth by the same factor. With Seedless the approach is different, as pulses can be calculated rapidly in a bespoke fashion based on the strength of the spectrometer's field, and a calibrated 90° pulse time that sets the field of the maximum amplitude. Although Seedless NMR pulses can also be rescaled as above if desired, it is better just to recalculate them on the fly.

In this section we compare the performance of bespoke Seedless pulses to a range of other GRAPE pulses that are available. Several different engines are available for generating NMR pulses using either GRAPE based approaches [25-27], and more, recently, AI based approaches [28]. The OC1.2 pulses are compared in a practical application to seedless pulses in **Supplementary Fig. 7** where we show that the performance of the two are very similar, but seedless allows for much shorter pulses to be calculated that perform the same function, that can lead to significant sensitivity gains.

In all cases, various restraints are imposed on pulses when they are calculated, including the number of bands, their frequencies, and the form of the amplitude inhomogeneity compensation. In what follows we have stated the conditions where the comparisons are made, matching as far as we can ascertain the conditions stated in the original papers. Based on the simulated infidelity scores, values obtained using Seedless pulses lower by factors in the range 2-10.

Where the improvement is a small factor (ca. 2), it is unlikely that the improvements will be obvious at the spectrometer whereas improvements on the order of 10 are likely to result in easily observable gains. The results illustrate that Seedless can very rapidly generate bespoke

pulses for any combination of restraints that are at least as good or even better than alternative approaches.

The additional convenience of Seedless is that it requires only the 90° calibrated pulse times to be entered, which will vary sample to sample, and pulses can always be matched to the sample. Because we need to accommodate the entire distribution of fields across the sample, so doing this will always be desirable to maximise sensitivity (**Main Text Fig. 1, Supplementary Fig. 2**).

Finally as described in the text, it is generally desirable to work with as long a time for each rectangular element in the pulse as possible[29], and so reduce transient response effects in the hardware. In all cases here, a Seedless pulse with a step duration of 2 μs achieved similar fidelities to those achieved with shorter times as used by the previously published pulses (**Supplementary Table 5**). Thus, pulses of similar performance can be achieved using Seedless using less stringent restraints. In all cases, rather than making one pulse to fit all occasions, superior pulses could be achieved by relaxing generality, and specialising the calculation to a specific application. As the Seedless calculation is so rapid, screening conditions to determine optimum input parameters can be easily and rapidly achieved. The performance of the pulses is summarised (**Supplementary Table 5**), and individual descriptions are provided (**Supplementary Note Figures 5.1-5**)

Source	Type	Step duration	Original Infidelity	Seedless Infidelity	Original/ Seedless
GeneticsAI[28]	Unitary 180°x (wide bandwidth)	380 ns	4.9 x10^{-3}	2.1 x10^{-3}	2.3
SURBOP [25]	Unitary 180°x	333ns	2.2 x 10^{-3}	7.6 x10^{-4}	2.9
GOODCOP [27]	$Z \rightarrow -Z$ (CO), $e^{-i\omega aTI_z}$ (CA)	200 ns	1.8 x10^{-3}	1.4 x 10^{-4}	12.8
BSCO(Ca)URBOP-180[26]	U180x (CO), Id (CA)	250 ns	9.2 x 10^{-3}	4.6 x 10^{-4}	2.0
		2000 ns		9.2 x10^{-3}	1.0

Supplementary Note Table 5 – A comparison of the performance of Seedless pulses to similar pulses in the literature. The calculated infidelity scores measure the performance of the pulses, and the ratio original/Seedless is a measure of the difference between the two methods. At the spectrometer, we would not expect to see a difference in fidelity of a small factor, but an order of magnitude or greater will likely lead to easily measurable effects in signal-to-noise. The Seedless pulses generated with identical restraints in all cases resulted in pulses with lower infidelity scores. We note that the duration of rectangular elements for the pulses (step duration) were typically short. Our general recommendation

for Seedless is to set this to a larger value to maximise the probability of the spectrometer exactly reproducing the effects of the *in-silico* calculation. In the case of the final pulse, the Seedless pulse infidelity achieved for a shorter (250 ns) and a longer pulse (2000 ns) element were highly similar. In all cases, Seedless matched the B_1 inhomogeneity compensation described in the original works. For the GENETICSAI method, a very wide distribution was supplied (+/- 15%), for SURBOP and GOODCOP values of +/-5% were supplied and for BSCO(Ca)URBOP-180° +/-20% was used.

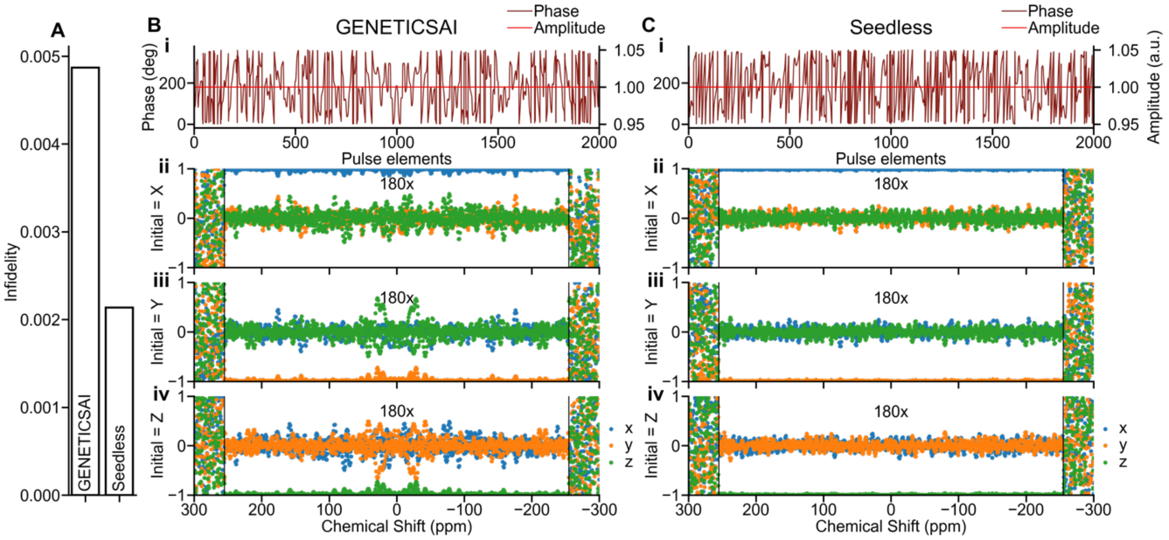

Supplementary Note Figure 5.1: A broadband unitary 180x pulse generated by GENETICSAI (**B** pix_76.000p1_bw12.28B1.mrf, link) [28] is compared with a Seedless pulse optimized under the same restraints (**C**), 306 kHz bandwidth (stated in the header of the GENETICSAI pulse), a peak field of 25 kHz and total duration of 760 µs, and amplitude inhomogeneity compensation using 0.95, 1.0 and 1.03 (we could not find the precise amplitude distribution used by the original authors). The infidelity of the GENETICSAI probe is high (4.9 x 10^{-3}) whereas the Seedless value is a factor of 2.3 lower (2.1 x 10^{-3}) (**A**). Performance is shown here for ^{1}H on a 600 MHz spectrometer, where the final state of the magnetization is shown following initialization on X (B/C ii), Y(B/C iii) or Z(B/C iv). The performance for all 3 fields considered in the calculation are shown. Substantially improved performance would be achieved by tuning the cost function to values closer to the required performance for the sample.

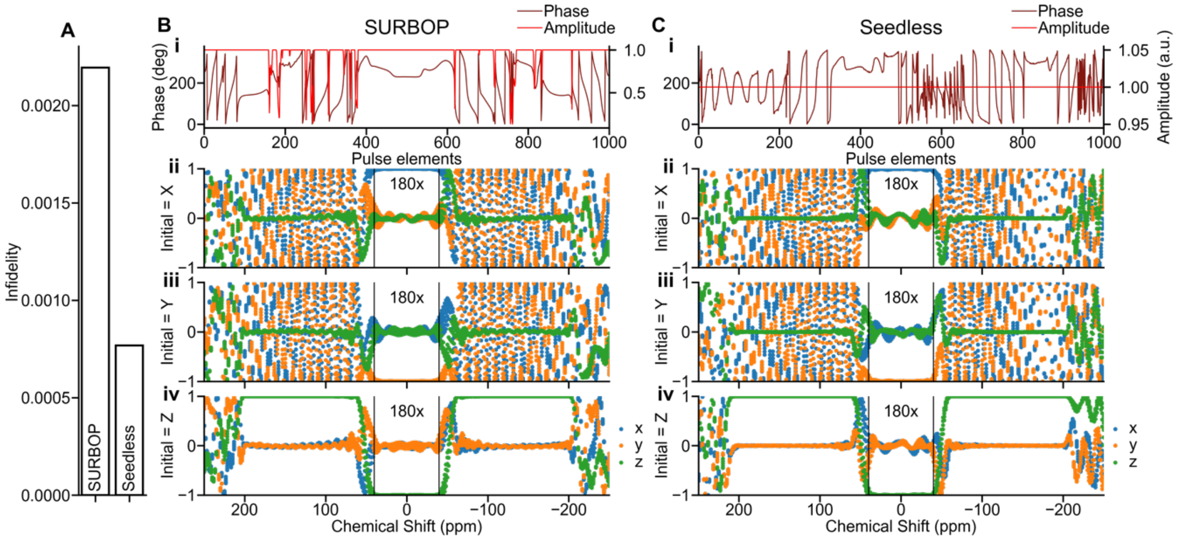

Supplementary Note Figure 5.2: A unitary 180°x pulse SURBOP pulse (**B –** SURBOP180 https://www.ioc.kit.edu/luy/111.php) [25] designed for application to ^{13}C on a 1.2 GHz spectrometer is compared with a Seedless equivalent (**C**). The SURBOP pulse has variable amplitude whereas Seedless is constant amplitude. The same restraints were used as stated for the SURBOP optimization, peak field of 15 kHz, duration 33.3 μs with 1000 points such that each element is 333 ns. Three chemical shift regions were defined: -200 to -60 ppm, -40 to +40 ppm and 60 to 200 ppm. 62, 31 and 62 frequencies were evenly spaced in these bands for both Seedless optimisation and infidelity calculation. The infidelity of the pulse, and the optimisation of the Seedless pulse was performed with an amplitude homogeneity compensation including 5 evenly weight relative fields (0.95, 0.975, 1.00, 1.025, 1.05). The infidelity of the Seedless pulse (7.6 x 10^{-4}) is a modest factor of 2.9 lower than the SURBOP pulse (2.2 x 10^{-3}) a difference unlikely to alter signal to noise at a spectrometer. (**A**). Performance is shown from initialising magnetization on X (**Bii**, **Cii**), Y (**Biii**, **Ciii**) and Z (**Biv**, **Civ**) and results for the five relative amplitudes considered in the calculation are shown.

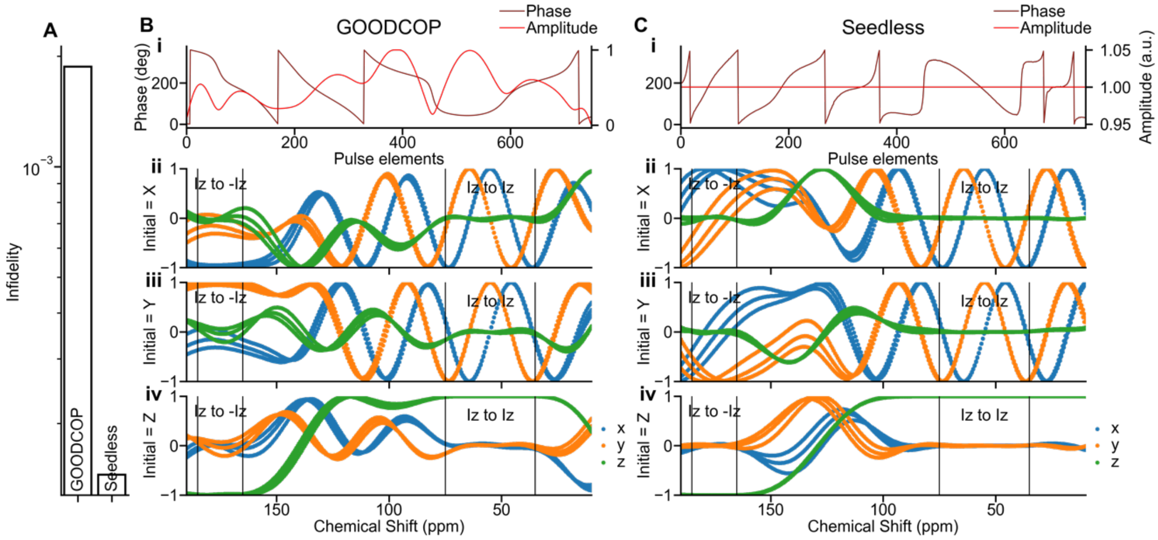

Supplementary Note Figure 5.3: The GOODCOP pulse (**B** - https://artlab.dana-farber.org/cocb-decoupling.html)[27] is equivalent to a Seedless pulse (**C**) designed to invert $Z \rightarrow -Z$ magnetisation for the Carbonyl region (165-185 ppm with 30 evenly spaced frequencies) while performing a unitary transformation ($e^{-i\omega a\,T\,I_z}$ with a = 0.9) on Cα/Cβ (35-75 ppm with 100 evenly spaced frequencies). The infidelity of the Seedless pulse (1.4 x 10^{-4}) is a factor of 12.9 lower than for GOODCOP (1.8 x 10^{-3}) (**A**). The differences in performance are visible in simulations, where magnetization is initialized on Z magnetisation (**Biv**, **Civ**). As stated in the header for the GOODCOP pulse, both pulses were set to 15 kHz maximum amplitude, the durations to 150 μs with 750 steps (each of 200 ns) and the carrier set to 55 ppm. Pulses were desired for 200 MHz (ie, ^{13}C on a 800 MHz spectrometer). We were unable to determine the specific B_1 tolerances used for the GOODCOP design, and so we used here our 3 measured relative amplitudes (0.95, 1.0 and 1.03), with both pulses showing similar (and good) tolerance to field inhomogeneity.

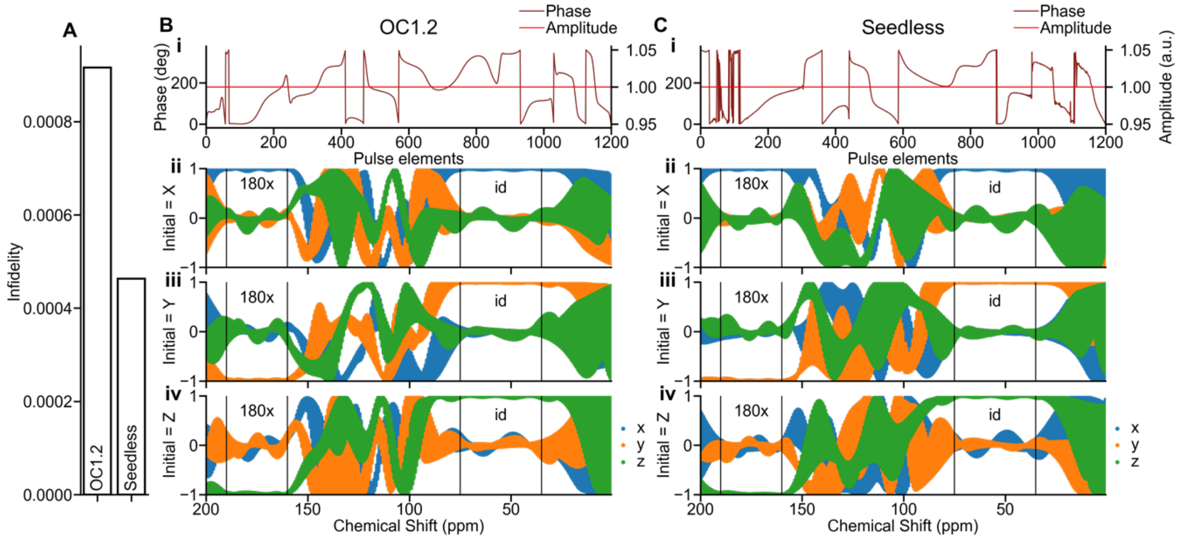

Supplementary Note Figure 5.4: Optimal control pulses for 1.2 GHz (OC1.2). An example is shown designed for 300 MHz field (^{13}C on a 1.2 GHz spectrometer) (**B** – named BSCO(Ca)URBOP-180° in the manuscript and BSCOURBOP_180deg_1200MHz_300us_RFA_15kHzB1d20per.dajo at link) [26] is shown here alongside a Seedless equivalent, optimised under as near to identical restraints as possible (**C**). Both were designed to perform a 180x Unitary on the Carbonyl carbons (originally stated as 9 kHz or 30 ppm – here defined as 160-190 ppm with 60 evenly spaced frequencies) and identity on Cα (originally stated as 12 kHz or 40 ppm – here defined as 35-75 ppm with 80 evenly spaced frequencies). We note the authors explicitly state their requirement of +/- 15% homogeneity on their 1.2 GHz spectrometer. The same field optimisations were used as in their original work, i.e. 121 evenly spaced frequencies with linear weighting. The resulting infidelity of the Seedless pulse (4.6×10^{-4}) is a factor of 2 lower than OC1.2 (9.2×10^{-4}) (**A**). As the pulses are close in value, it is unlikely that the differences will amount to any substantial differences in signal-to-noise levels in real spectra. Due to low pulse element duration (250 ns) and fine sampling of field distribution, this calculation took several minutes on a Macbook Pro with M1 Pro, whereas OC1.2 takes several hours using Spinach[26]. We demonstrate that more typical Seedless parameters enable calculation of a pulse with equivalent fidelity to OC1.2 in 12.5 seconds (**Supplementary Fig. 6.5**). Performance is

shown following initializing magnetisation on X (**Bii**, **Cii**), Y (**Biii**, **Ciii**) and Z (**Biv**, **Civ**). The maximum fields for both pulses were set to 15 kHz, the durations to 300 μs and the carriers to 100 ppm (as required by the OC1.2 pulse).

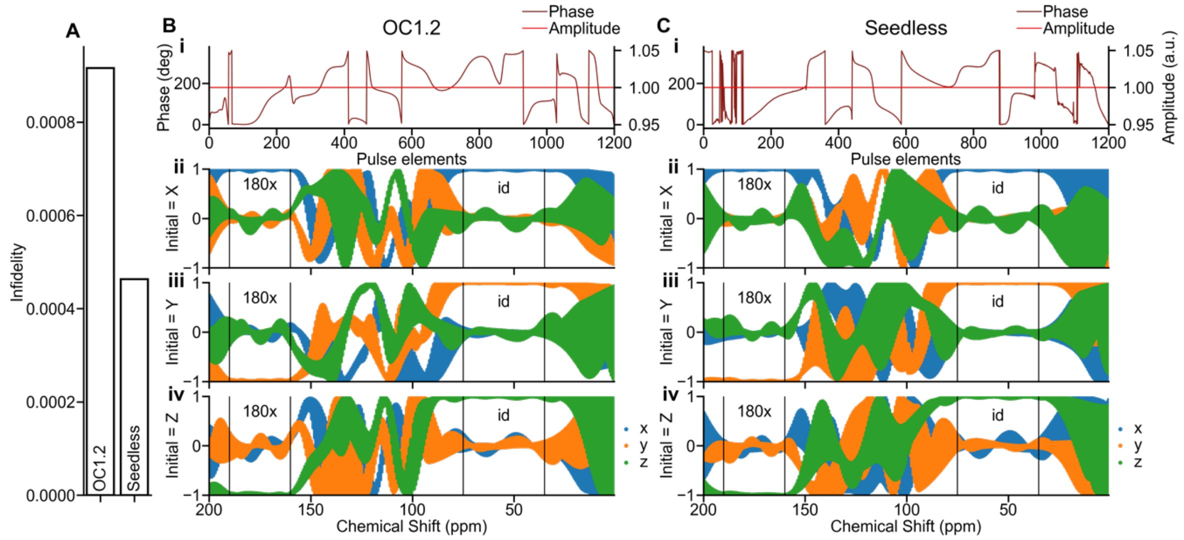

Supplementary Note Figure 5.5: Seedless can generate a pulse with near identical infidelity with far fewer restraints. With a large number (121) of field values, and a short single element duration (250 ns) for OC1.2, (**Supplementary Note Figure 5.4)** we sought to compute a pulse for the same overall action, but using restraints with have otherwise worked with in this work using Seedless pulses: 3 relative field values at 0.8, 1.0 and 1.2 weighted equally, and a 2 μs rectangular element duration, a calculation taking 12.5 seconds. The resulting fidelity (calculated under the same restraints as in **Supplementary Note Figure 5.4**) results in a pulse with equivalent fidelity to OC1.2 (9.2 x 10^{-4} for both).

Supplementary References

1 Bhole, G. & Jones, J. A. Practical pulse engineering: Gradient ascent without matrix exponentiation. *Frontiers of Physics* **13**, 130312, doi:10.1007/s11467-018-0791-1 (2018).

2 Jones, J. A. Controlling NMR spin systems for quantum computation. *Progress in Nuclear Magnetic Resonance Spectroscopy* **140-141**, 49-85, doi:https://doi.org/10.1016/j.pnmrs.2024.02.002 (2024).

3 Khaneja, N., Reiss, T., Kehlet, C., Schulte-Herbruggen, T. & Glaser, S. J. Optimal control of coupled spin dynamics: design of NMR pulse sequences by gradient ascent algorithms. *J Magn Reson* **172**, 296-305, doi:10.1016/j.jmr.2004.11.004 (2005).

4 Jozsa, R. Fidelity for Mixed Quantum States. *Journal of Modern Optics* **41**, 2315-2323, doi:Doi 10.1080/09500349414552171 (1994).

5 Baldwin, A. J. & Jones, J. A. Efficiently computing the Uhlmann fidelity for density matrices. *Physical Review A* **107**, 012427, doi:10.1103/PhysRevA.107.012427 (2023).

6 Glaser, S. J. *et al.* Unitary control in quantum ensembles: maximizing signal intensity in coherent spectroscopy. *Science* **280**, 421-424, doi:10.1126/science.280.5362.421 (1998).

7 Bloch, F. & Siegert, A. Magnetic resonance for nonrotating fields. *Physical Review* **57**, 522-527, doi:DOI 10.1103/PhysRev.57.522 (1940).

8 Ramsey, N. F. Resonance Transitions Induced by Perturbations at Two or More Different Frequencies. *Physical Review* **100**, 1191-1194, doi:10.1103/PhysRev.100.1191 (1955).

9 Chuang, I. L., Gershenfeld, N., Kubinec, M. G. & Leung, D. W. Bulk quantum computation with nuclear magnetic resonance: theory and experiment. *Proceedings of the Royal Society of London. Series A: Mathematical, Physical and Engineering Sciences* **454**, 447-467, doi:10.1098/rspa.1998.0170 (1998).

10 Cory, D. G., Fahmy, A. F. & Havel, T. F. Ensemble quantum computing by NMR spectroscopy. *Proc Natl Acad Sci U S A* **94**, 1634-1639, doi:10.1073/pnas.94.5.1634 (1997).

11 Jones, J. A. Designing short robust not gates for quantum computation. *Physical Review A* **87**, 052317, doi:10.1103/PhysRevA.87.052317 (2013).

12 Bowdrey, M. D., Oi, D. K. L., Short, A. J., Banaszek, K. & Jones, J. A. Fidelity of single qubit maps. *Physics Letters A* **294**, 258-260, doi:Pii S0375-9601(02)00069-5

13 de Fouquieres, P., Schirmer, S. G., Glaser, S. J. & Kuprov, I. Second order gradient ascent pulse engineering. *J Magn Reson* **212**, 412-417, doi:10.1016/j.jmr.2011.07.023 (2011).

14 Rabitz, H. A., Hsieh, M. M. & Rosenthal, C. M. Quantum optimally controlled transition landscapes. *Science* **303**, 1998-2001, doi:10.1126/science.1093649 (2004).

15 Violaris, M., Bhole, G., Jones, J. A., Vedral, V. & Marletto, C. Transforming pure and mixed states using an NMR quantum homogenizer. *Physical Review A* **103**, 022414, doi:10.1103/PhysRevA.103.022414 (2021).

16 Luy, B., Kobzar, K., Skinner, T. E., Khaneja, N. & Glaser, S. J. Construction of universal rotations from point-to-point transformations. *Journal of Magnetic Resonance* **176**, 179-186, doi:https://doi.org/10.1016/j.jmr.2005.06.002 (2005).

17 Husain, S., Kawamura, M. & Jones, J. A. Further analysis of some symmetric and antisymmetric composite pulses for tackling pulse strength errors. *Journal of Magnetic Resonance* **230**, 145-154, doi:https://doi.org/10.1016/j.jmr.2013.02.007 (2013).

18 Emsley, L. & Bodenhausen, G. Optimization of shaped selective pulses for NMR using a quaternion description of their overall propagators. *Journal of Magnetic Resonance (1969)* **97**, 135-148, doi:https://doi.org/10.1016/0022-2364(92)90242-Y (1992).

19 Liu, D. C. & Nocedal, J. On the limited memory BFGS method for large scale optimization. *Mathematical Programming* **45**, 503-528, doi:10.1007/BF01589116 (1989).

20 Nocedal, J. Updating Quasi-Newton Matrices with Limited Storage. *Mathematics of Computation* **35**, 773-782, doi:10.2307/2006193 (1980).

21 Moré, J. J. & Thuente, D. J. Line search algorithms with guaranteed sufficient decrease. *ACM Trans. Math. Softw.* **20**, 286–307, doi:10.1145/192115.192132 (1994).

22 de Fouquieres, P., Schirmer, S. G., Glaser, S. J. & Kuprov, I. Second order gradient ascent pulse engineering. *Journal of Magnetic Resonance* **212**, 412-417, doi:https://doi.org/10.1016/j.jmr.2011.07.023 (2011).

23 Buek, V., Hillery, M. & Werner, F. Universal-NOT gate. *Journal of Modern Optics* **47**, 211-232, doi:10.1080/09500340008244037 (2000).

24 Kay, L. E., Ikura, M., Tschudin, R. & Bax, A. Three-dimensional triple-resonance NMR spectroscopy of isotopically enriched proteins. *Journal of Magnetic Resonance (1969)* **89**, 496-514, doi:https://doi.org/10.1016/0022-2364(90)90333-5 (1990).

25 Slad, S., Bermel, W., Kümmerle, R., Mathieu, D. & Luy, B. Band-selective universal 90° and 180° rotation pulses covering the aliphatic carbon chemical shift range for triple resonance experiments on 1.2 GHz spectrometers. *J Biomol NMR* **76**, 185-195, doi:10.1007/s10858-022-00404-1 (2022).

26 Joseph, D. & Griesinger, C. Optimal control pulses for the 1.2-GHz (28.2-T) NMR spectrometers. *Science Advances* **9**, eadj1133, doi:10.1126/sciadv.adj1133.

27 Coote, P. W. *et al.* Optimal control theory enables homonuclear decoupling without Bloch–Siegert shifts in NMR spectroscopy. *Nature Communications* **9**, 3014, doi:10.1038/s41467-018-05400-4 (2018).

28 Subrahmanian, M. V., Pavuluri, K., Olivieri, C. & Veglia, G. High-fidelity control of spin ensemble dynamics via artificial intelligence: from quantum computing to NMR spectroscopy and imaging. *PNAS Nexus* **1**, pgac133, doi:10.1093/pnasnexus/pgac133 (2022).

29 Tabuchi, Y., Negoro, M., Takeda, K. & Kitagawa, M. Total compensation of pulse transients inside a resonator. *Journal of Magnetic Resonance* **204**, 327-332, doi:https://doi.org/10.1016/j.jmr.2010.03.014 (2010).

3 pulse types:

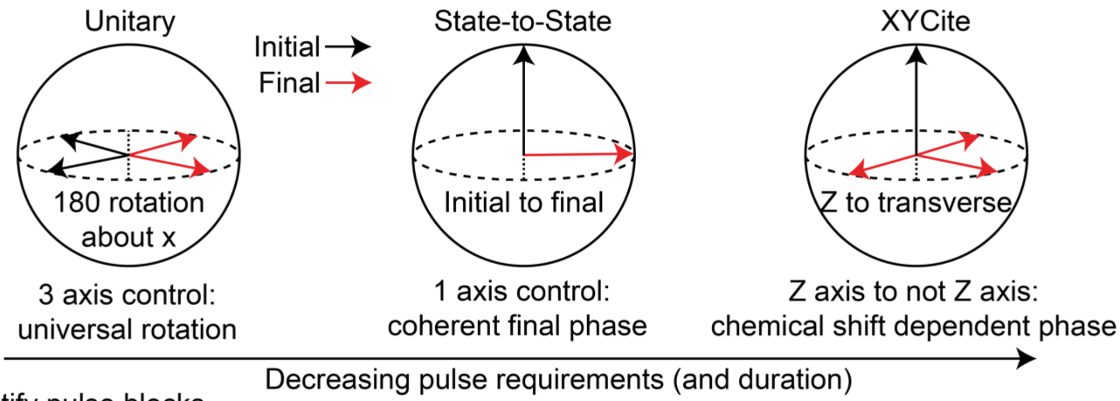

1. Identify pulse blocks

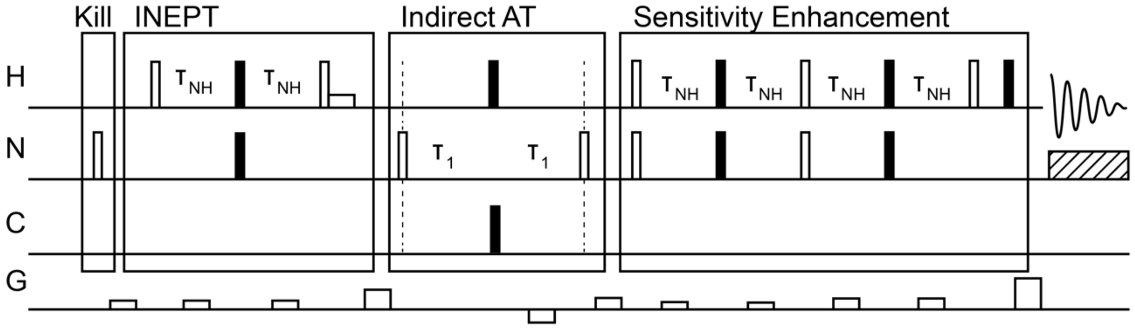

2. Identify pulse requirements

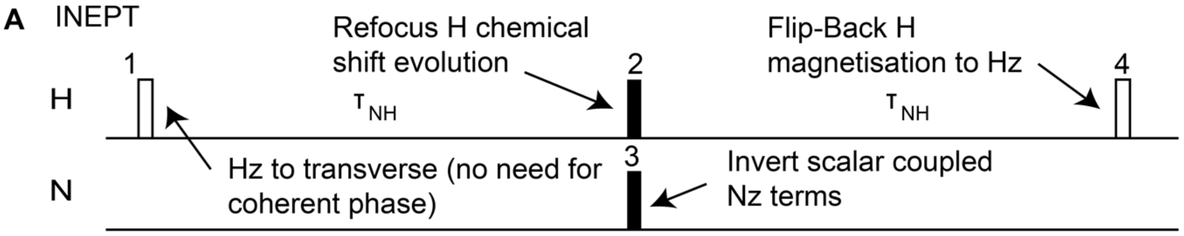

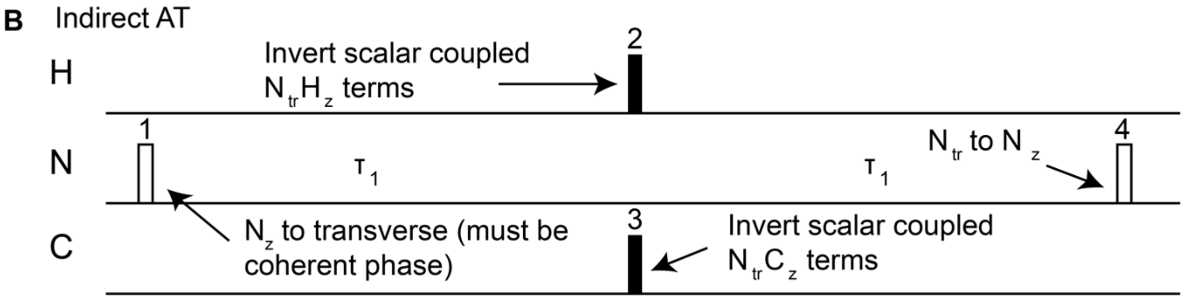

3. Choose pulse types:

**A**	1 = Hz -> -Hy<br>or XCite<br>or 90x unitary	2 = Unitary 180	3 = Nz -> -Nz<br>or Unitary 180	4 = Hy -> Hz<br>or time-rev XCite<br>or 90x unitary
**B**	1 = N_z -> $-N_y$<br>or 90x unitary	2 = H_z -> $-H_z$<br>or Unitary 180	3 = C_z -> $-C_z$<br>or Unitary 180	4 = N_y -> N_z<br>or 90x unitary

Supplementary Fig. 1: Graphical overview of seedless pulses. Seedless can design chemical shift bands to perform universal rotations (**Ai**, 3 axis control), state-to-state pulses that perform 1 axis control (**Aii**), or XYcite pulses that move magnetization from the longitudinal axis to the transverse plane, but without coherent control in the XY plane (**Aiii**) (**Table 1, Supplementary Table 2.1**). For the same infidelity, pulses that control more axes will require a longer durations / higher amplitude field. Where relaxation is a problem, and where hardware specifications are restrictive, shorter pulses are desirable, and so design should be limited to the amount of control required. To identify what is required, the functions of individual pulses in a sequence first need to be identified (**B, C**). For 180° pulses acting on transverse magnetization (refocusing), the pulse must be universal. Where initial and final states are well defined (e.g. $\mathrm{Z} \rightarrow -\mathrm{Y}$ excitation and $\mathrm{Y} \rightarrow \mathrm{Z}$, de-excitation), a state-to-state restraint is sufficient and while a universal rotation would also work, it is not necessary. Where coherent phase ordering in the XY plane is not required, such as during an INEPT transfer, an XYcite pulse can be used. Specific considerations applied within an HSQC for the INEPT transfer (A) and indirect chemical shift acquisition (B) are shown. During a refocused INEPT in a sensitivity enhanced or TROSY experiment, the ^{15}N 90° pulses need to handle control over multiple axes, and so need to be unitary.

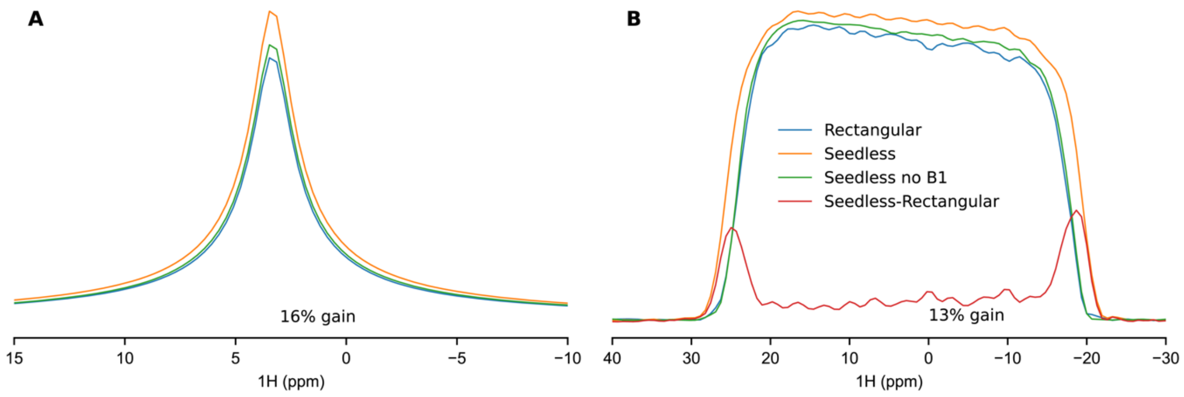

Supplementary Fig. 2: B_1 inhomogeneity compensation revealed using imaging. The ^{13}C HSQC imaging experiment (**Supplementary Note 3.1**) was performed on a Z-gradient equipped triple resonance inverse probe both without (**A**) and with (**B**) gradient echo applied during acquisition for imaging, a pulse sequence using rectangular pulses (blue), Seedless pulses (orange), and Seedless pulses with amplitude compensation removed (green). All spectra are shown in absolute value mode, analysing the $^{13}C/^{1}H$ resonance from ^{13}C enriched methanol (**methods**), and the difference between Seedless and rectangular (orange-blue) is shown in red. Comparing the B_1 optimised Seedless pulse (orange) to the rectangular (blue) reveal 16% (**A**) 13% (**B**) gains using Seedless. Notably, when Seedless pulses without B_1 compensation were used, these improvements were mostly removed (green). As there is only one gradient axis on this probe, coherence selection gradients had to be applied on the same axis as imaging gradients, which resulted in small oscillating fluctuations in the imaging profile (**B**). These artefacts were removed when we separated selection and imaging gradients on the 5 mm three channel XYZ-gradient probe (**Main Text Fig. 1A**). The sensitivity gains on the two probes were similar, as were the Z-profiles indicating that B_1 homogeneity correction is effectively increasing the coil volume by compensating for divergence in the excitation profiles produced by the saddle coils used for RF generation.

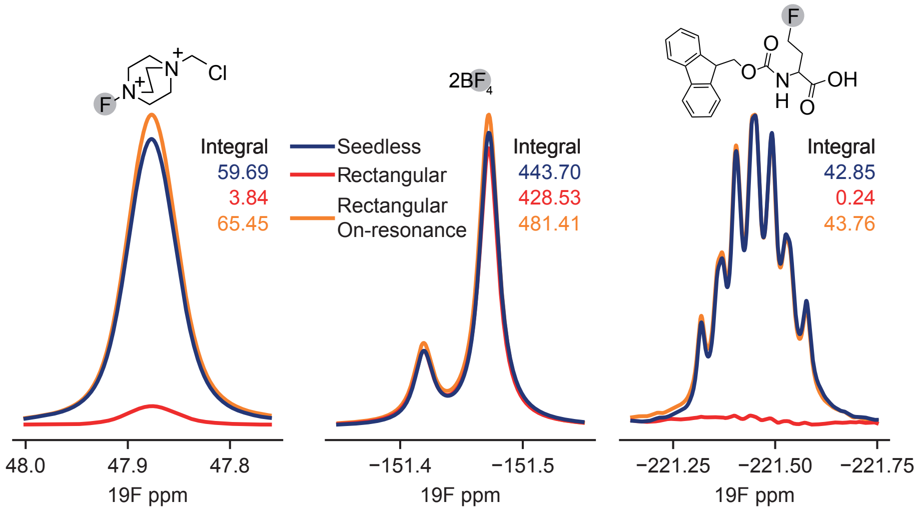

Supplementary Fig. 3: Detailed comparison of Seedless ^{19}F ultrabroadband experiment. This was tested on a sample containing Selectfluor together with its BF_4^- counterion and an Fmoc fluorinated amino acid in a 1:1 molar ratio, prepared as described (**methods**). Five experiments were run, Seedless (Blue), and four with rectangular pulses , one centred at the central frequency -82 ppm (red), and three centred on each of the species at 47.89 ppm (orange, left), -151.5 ppm (orange, middle) and -221.5 ppm (orange, right). In all cases, the overall integrated intensity was marginally lower in the Seedless pulses than the on-resonance rectangular pulses, reflecting the Seedless pulse duration being 2000 μs versus 12.6 μs for the rectangular pulses. However, the Seedless pulse simultaneously excites all three species in one experiment, providing quantifiable spectra without baseline distortions requiring only 0th order phase correction. By contrast, the spectrum achieved with a single rectangular pulse centred at -82 ppm cannot make all resonances into a pure absorption mode using only 0th and 1st order phase corrections and the intensities of the species far from the carrier at 47.89 ppm and -221.5 are decreased by factors of 16 and 178 respective to the Seedless spectrum owing to the limited excitation profile of the short rectangular pulse (**Main Text Fig. 1**). The expected ratio for BF_4^- to the organic component of selectfluor was 7.3 for the on-resonance rectangular pulses and 7.5 for Seedless, which is closer to the expected value of 8.

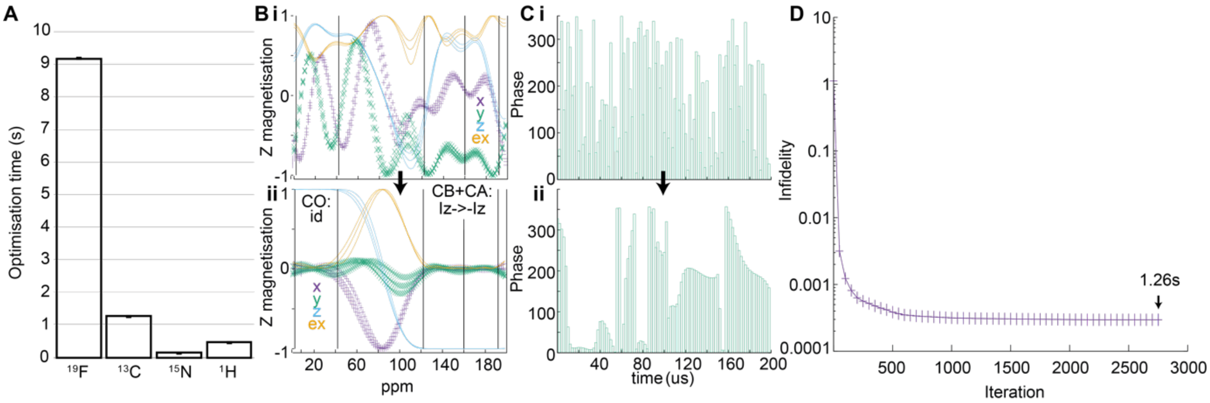

Supplementary Fig. 4: Monitoring progress of a seedless optimisation. The Seedless calculation time for 4 example pulses used in this manuscript varies in the region of ca. 0.15 s / 0.5 s / 1.2 s for ^{15}N/^{1}H/^{13}C (for ^{15}N HSQC and the triple resonance experiments **Main Text Figs. 2B,3** and **5**, **Supplementary Notes 3.4-8** where ^{13}C pulses are band selective and ^{15}N/^{1}H are not), to 9.17 s (**A**, the broadband ^{19}F pulse, **Main Text Fig. 1B, Supplementary Note 3.2**). These tests were performed on a 2021 Macbook pro with a 10 core M1 Pro processor and 16GB of RAM with the number of CPUs set to 8. An example ^{13}C pulse optimisation is shown (**B-D**). This specific pulse is designed to perform a state-to-state $Z \rightarrow -Z$ inversion on the Cα and Cβ spins, alongside an identity (id) operation on CO spins (**B**). This is used in the HNCO pulse sequence to refocus Cα/Cβ coupling during indirect acquisition (pulse **iii** for the HNCO, **Supplementary Note 3.5**). The pulse is started from random phases (**Ci**), arriving at the final pulse after 2,700 iterations in 1.26 s. Typically the infidelity falls quickly to a state where diminishing returns are found with the number of iterations. In this case coverage was achieved by the difference in infidelity being less than 10^{-10} (default value of epsilon, **Supplementary Note 5**).

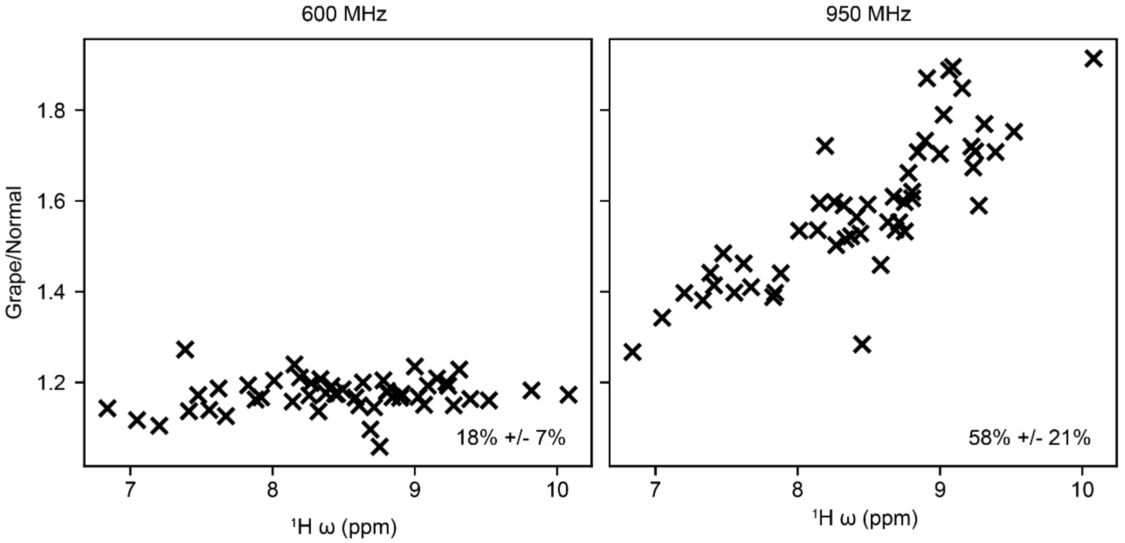

Supplementary Fig. 5: Comparison of 600 MHz and 950 MHz ^{15}N HSQC data Seedless/rectangular. Experiments were performed on a sample of U[^{1}H/^{15}N/^{13}C] ABP1P acquired at 600 and 950 MHz, supporting **Main Text Fig. 2**. The ratios of intensities from the Seedless and rectangular pulse sequences from sensitivity enhanced HSQCs (**Supplementary Note 3.4**) at each field are shown for each resonance as a function of the amide proton chemical shift. On a 5 mm HCN room-temperature probe at 600 MHz, no obvious trend with chemical shift is observed, suggesting that the average gains of 18% are due purely to the B_1 inhomogeneity compensation. By contrast, on a 5 mm TCI cryogenically cooled probe at 950 MHz, an approximately linear correlation is observed with proton chemical shift indicating that the B_1 inhomogeneity compensation, and improved bandwidth of the pulses are both contributing to the 58% average gains with more gains at higher ^{1}H ppm values. The 90° ^{1}H rectangular pulse times were 10.7 μs (600 MHz) and 14.2 μs (950 MHz) respectively indicating peak fields of 23.4 kHz and 17.6 kHz. The increase in 90° time on the cryo-probe reflected the relatively high salt buffer used (PBS, **methods**). Achieving uniform wide bandwidth excitation is expected to become more challenging as the field of the spectrometer increases.

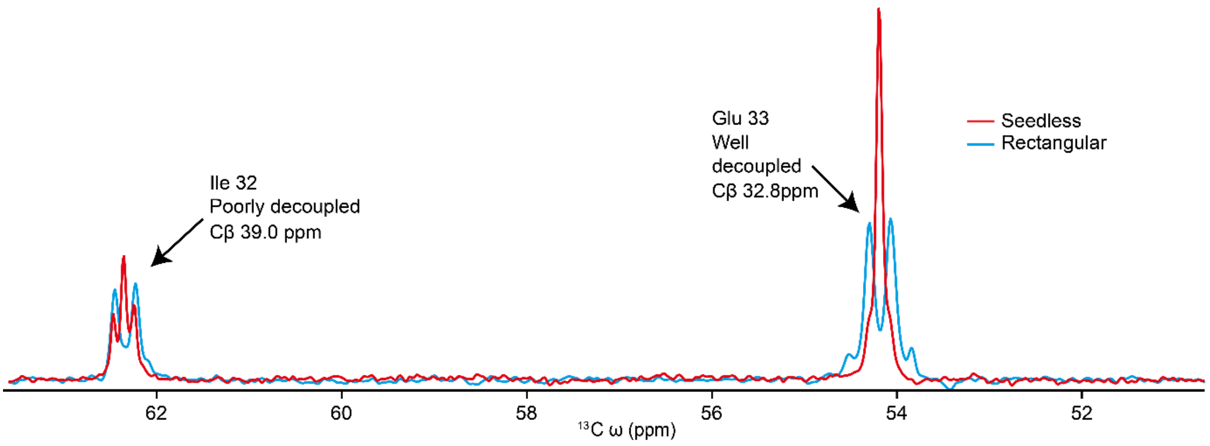

Supplementary Fig. 6: Cα -Cβ Decoupling. Coupling between the Cα and Cβ carbon atoms can lead to line broadening in observed signals when Cα is detected, as occurs in the HNCA and HNCOCA experiments (**Supplementary Notes 3.6/8**) and during the Cα→CO transfer when Cα is transverse in the HNCACO (**Supplementary Note 3.7**). In these cases, pulses are required that treat the Cα and Cβ bands differently as described in the text, methods and **Supplementary Note 3**. As the Cα distribution ranges in proteins approximately from 40 to 70 ppm, Cβs for serine and threonine residues are between 60 to 80 ppm, and for all other residues in the range 15 to 50 ppm (**Main Text Fig. 3A**), it is impossible in general using one-spin methods to perfectly distinguish Cα/Cβ atoms in a single experiment and compromises must be made. Pulses to separate Cα/Cβ were calculated here with a Cα/Cβ interface set to 36 ppm, a total pulse duration 400 μs at a field of 17.6 kHz, designed for application at 600 MHz (**Supplementary Note 3**). With these settings, the 'interface' region with unpredictable performance, between where Cα and Cβ were controlled was achieved at 36 +/- 4 ppm (**Supplementary Notes 3.6/7/8, pulse iii**). The effects of this are seen clearly in a slice from an HNCA that has high ^{13}C resolution (70 complex points sampled in the range 0-249 for a 2250 Hz sweep width, giving a 9 Hz resolution). When using rectangular pulses only, coupling is evident (blue). Using Seedless pulses (red) perfect decoupling of most residues is achieved, including Glu 33 (Cα 54 ppm, Cβ 32.8 ppm, right). By contrast, for Ile 32 at (Cα 62.3 ppm, Cβ 39.0 ppm), the Cβ sits within the 'interface' region and so is only partially decoupled (left). Resonances from all Ser/Thr residues are fully coupled (not shown), but pulses can be

designed where this is required. Using Seedless, pulses can be trivially constructed with performance matches exactly to the sample / hardware. Notably, increasing the peak B_1 and overall duration will reduce the window region where Cα/Cβ control is impaired and multiple spectra could in principle be acquired moving the Cα/Cβ interfaces if Cβ decoupling is absolutely required. For ease of analysis of triple resonance experiments recording a Cα dimension (HNCA/HNCOCA), we recommend the conventional wisdom of recording at lower resolution acquisition to prevent resolution of the Cβ coupling (typically 45 Hz resolution, set to be slightly larger than the expected Cα-Cβ J coupling, 28-40 Hz). In this case, sensitivity improvements for most residues are still achieved via imperfect Cβ decoupling as described above but the coupling is not resolved. Similarly, with the HNCACO, significant sensitivity enhancements were obtained via Cβ decoupling during the Cα-CO transfer regions, but we empirically determine that it is more important to achieve excellent Cα control with imperfect Cβ decoupling to maximise overall sensitivity gains.

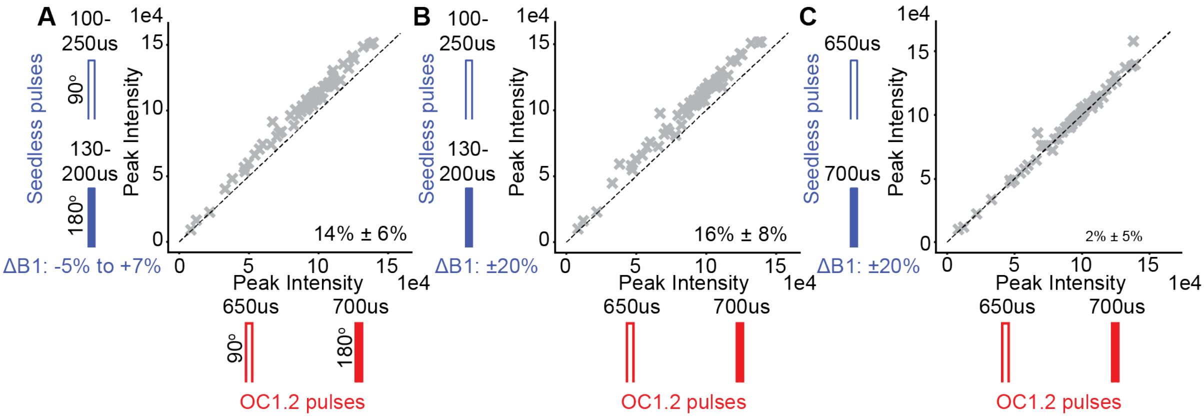

Supplementary Fig. 7: Practical comparison on OC1.2 and seedless pulses. Pre-calculated shaped pulses are used in NMR with either duration and/or peak B_1 field rescaled to fit specific applications. We sought to compare recently published constant amplitude OC pulses optimised for a 1.2 GHz NMR spectrometer [26] to those created by Seedless in a ^{15}N HSQC experiment. We used ^{1}H and ^{15}N OC1.2 90° (650 μs, A, B, C, red, open rectangle) and 180° (700 μs, A, B, C, red, filled rectangle) degree Unitary pulses to create the pulse sequence, applied at a ^{1}H field of 8 kHz and a ^{15}N field of 5 kHz, as described in the original work[26]. We created Seedless pulses for ^{1}H and ^{15}N according to our hardware specifications, resulting in pulses of total duration between 100 and 250 μs applied at a ^{1}H field of 23.8 kHz and ^{15}N field of 7.35 kHz (**Supplementary Note 3.4**). For simplicity, the carbon refocusing pulse during indirect acquisition was a 200 μs Seedless 180-degree universal pulse at a field of 17.1 kHz in both experiments. Both sets of pulses were implemented on a 600 MHz RT NMR spectrometer and we analysed the abp1p protein at 25°C.

A – A 14% average sensitivity enhancement by using Seedless pulses, versus the OC1.2 pulses. **B –** The inhomogeneity distribution used for the OC1.2 pulses was broader than that which we typically used for Seedless. We re-created Seedless pulses with a B_1 inhomogeneity distribution of 0.8, 1.0, 1.2 weighed at 0.25, 0.5, 0.25 to make a better match. We observed a 16% average sensitivity enhancement. **C –** we repeated the test in (A) but using Seedless pulses whose field/duration match those of the OC1.2 pulses. We saw essentially the same signal in both cases.

The total pulse duration for the OC1.2 pulses summed over the pulse sequence was 8.1 ms, whereas for Seedless the total pulse duration was 2.15 ms (**A/B**). An average relaxation rate over the pulse sequence of 25 s^{-1} is sufficient to explain the 14% intensity difference, which is a reasonable value for a small globular protein such as abp1p. The sensitivity gains in this case come simply from swapping longer pulses (OC1.2) with shorter pulses (Seedless).

Taken together, we conclude 1) Seedless pulses are very similar in fidelity and performance to those optimised under similar restraints, the difference being Seedless pulses take a few seconds per calculation rather than tens of hours and 2) tailoring the pulse to the specific sample / hardware combination can deliver substantial sensitivity gains, and that 'one size does not fit all'.
